# Every contact leaves a trace: Documenting contamination in lithic residue studies at the Middle Palaeolithic sites of Lusakert Cave 1 (Armenia) and Crvena Stijena (Montenegro)

**DOI:** 10.1371/journal.pone.0266362

**Published:** 2022-04-07

**Authors:** Ellery Frahm, Daniel S. Adler, Boris Gasparyan, Bing Luo, Carolina Mallol, Goran Pajović, Gilbert B. Tostevin, Benik Yeritsyan, Gilliane Monnier

**Affiliations:** 1 Department of Anthropology, Yale University, New Haven, Connecticut, United States of America; 2 Department of Anthropology, University of Connecticut, Storrs, Connecticut, United States of America; 3 Institute of Archaeology and Ethnography, National Academy of Sciences of the Republic of Armenia, Yerevan, Armenia; 4 Characterization Facility, University of Minnesota, Minneapolis, Minnesota, United States of America; 5 Departamento de Prehistoria, Antropología e Historia Antigua, Universidad de La Laguna, La Laguna, Tenerife, Spain; 6 National Museum of Montenegro, Cetinje, Montenegro; 7 Department of Anthropology, University of Minnesota, Minneapolis, Minnesota, United States of America; Universita degli Studi di Ferrara, ITALY

## Abstract

Investigations of organic lithic micro-residues have, over the last decade, shifted from entirely morphological observations using visible-light microscopy to compositional ones using scanning electron microscopy and Fourier-transform infrared microspectroscopy, providing a seemingly objective chemical basis for residue identifications. Contamination, though, remains a problem that can affect these results. Modern contaminants, accumulated during the post-excavation lives of artifacts, are pervasive, subtle, and even “invisible” (unlisted ingredients in common lab products). Ancient contamination is a second issue. The aim of residue analysis is to recognize residues related to use, but other types of residues can also accumulate on artifacts. Caves are subject to various taphonomic forces and organic inputs, and use-related residues can degrade into secondary compounds. This organic “background noise” must be taken into consideration. Here we show that residue contamination is more pervasive than is often appreciated, as revealed by our studies of Middle Palaeolithic artifacts from two sites: Lusakert Cave 1 in Armenia and Crvena Stijena in Montenegro. First, we explain how artifacts from Lusakert Cave 1, despite being handled following specialized protocols, were tainted by a modern-day contaminant from an unanticipated source: a release agent used inside the zip-top bags that are ubiquitous in the field and lab. Second, we document that, when non-artifact “controls” are studied alongside artifacts from Crvena Stijena, comparisons reveal that organic residues are adhered to both, indicating that they are prevalent throughout the sediments and not necessarily related to use. We provide suggestions for reducing contamination and increasing the reliability of residue studies. Ultimately, we propose that archaeologists working in the field of residue studies must start with the null hypothesis that miniscule organic residues reflect contamination, either ancient or modern, and systematically proceed to rule out all possible contaminants before interpreting them as evidence of an artifact’s use in the distant past.

## Introduction

The allure of identifying microscopic residues left behind on lithic artifacts is undeniable. Generations of archaeologists have construed the uses of particular stone tool types on the basis of an intuitive interpretation of their shapes, by analogy with more recent tools (e.g., scrapers, awls, knives, sickles), or on the basis of often controversial use-wear analyses. Empirical data enabling the reconstruction of lithic artifact functions, therefore, offers an attractive means of reconstructing human behavior with greater nuance and reliability and with less inference. Archaeologists using visible-light microscopy (VLM) have reported finding microscopic residues related to the processing of plants (e.g., starch grains, plant fibers, wood) and animals (e.g., collagen, bone, fat, hair, feathers, scales) adhering to lithic artifacts [1–6]. Residue identification via VLM, though, is confounded by a variety of issues, including morphological alteration during use and degradation during burial [7–9] as well as the experience level and subjective interpretation of the observer, as revealed through blind tests [9]. For example, Croft et al. [10] established that, when compared to spectroscopic data, all residues on lithic artifacts from the Mesolithic site of Star Carr (England) had been misidentified via VLM (e.g., gypsum was identified as wood fibers or silica plant phytoliths, iron oxide was identified as resin). Additionally, there are few, if any, standards for reporting or documenting visual traits of residues, adding to an inherent subjectivity.

To minimize these issues, we have focused on the use of reflectance Fourier-transform infrared microspectroscopy (μFTIR), supplemented by scanning electron microscopy (SEM), to document plant- and animal-tissues on experimental lithic artifacts [11, 12] with the intent to apply these techniques to artifacts from newly excavated Palaeolithic assemblages. Previously, Monnier et al. [13] employed μFTIR and SEM to chemically identify macroscopic patches of bitumen adhered to Levallois points from the Middle Paleolithic site of Hummal (Syria). Others have similarly utilized μFTIR and/or SEM to examine experimental artifacts [14–16] or to report the occurrence and identifications of apparent use-related residues adhered to Pleistocene artifacts [17–20]. These studies have demonstrated that μFTIR provides a means of avoiding the risk of connoisseurship that is arguably inherent to VLM observations by providing a chemical basis to both describe and document residues in a standardized way.

Contamination is an issue, however, that plagues VLM, SEM, and μFTIR alike. Modern sources of contamination, deposited on artifacts during their post-excavation histories, are pervasive and must be given careful, critical consideration [16, 21]. One well-documented example is the use of powder-coated examination gloves to handle artifacts, which contaminates them with modern starch grains [22]. Other potential sources of contamination are much more subtle. Most readers have likely seen lithic artifacts stored or transported in wooden drawers, burlap sacks, rice bags, or cardboard boxes. Even the artifacts carefully curated in museums’ cabinet drawers come into contact with, for example, various foams that surround their edges. Rarely is the complete storage history for an assemblage documented. The same artifacts may also have been extensively handled during cleaning, perhaps involving implements such as a plastic toothbrush, a wood spatula or skewer, or even metal dental tools. Some artifacts are illustrated and analyzed before being subjected to residue studies, meaning that they have been extensively handled or even in direct contact with paper and/or pencils. One of us (GM) was once asked to identify residues on sickle blades, which turned out to be the plastic clay into which the artifacts had been pressed as a means to stabilize them under the microscope for use-wear analysis. Although this example is especially egregious, it has been documented that simply handling artifacts with bare hands can deposit skin flakes, oils from skin’s sebaceous glands, and sweat, which contains salt, amino acids, and even proteins [21]. Furthermore, personal care products (e.g., hand lotion, sunscreen) that may be applied to the analyst’s hands contain a variety of ingredients that can be transmitted to artifacts’ surfaces. Some archaeologists report that artifacts subjected to residue studies were washed (e.g., “archaeological specimens were only washed in fresh water in order to remove the soil deposits and finger grease resulting from touching their surfaces,” 17). While it is true that some of the potential contaminants are water-soluble and might be removed by rinsing the artifacts with water, other substances are not and can remain adhered even after ultrasonic cleaning in a bath of distilled or deionized water. In short, there are many stages during which contaminants can be introduced, and removing them without damaging potential use-related residues requires careful consideration. Consequently, it is greatly concerning to read studies in which microscopic residues were found on artifacts only after their extensive handling for classification, techno-typological analysis, illustration, photography, etc. and/or were stored in unreported conditions.

Ancient contamination is another issue with which researchers must contend. The goal of lithic residue analysis is to identify any residues directly associated with human use of the artifacts; however, other types of residues can also accumulate on surfaces post-depositionally. Residues can accumulate as a result of various site-formation and diagenetic processes (e.g., bioturbation, pedogenesis, weathering), and use-related residues can also degrade into secondary products (e.g., replacement of a residue by fungal hyphae; 8). Other situations can be envisioned, such as trampling by humans or animals that could potentially lead to abrasions between lithic artifacts and bone fragments deposited together. Given the potential of residues to offer insights into Pleistocene hominin behaviors, Palaeolithic sites in caves have been a particular focus of interest for archaeologists (e.g., Qesem Cave [17, 20]). Caves are commonly regarded as more “protected” environments than open-air sites, but they are still subject to a range of taphonomic forces and geo-, bio-, and anthropogenic inputs [23–25]. Considerable amounts of organic material can accumulate in the form of human, bat, bird, and carnivore (e.g., bears, hyenas) excrement and remains, for example. Organic compounds can become oxidized or decay, while minerals, including carbonates, phosphates, and silicates, can dissolve, transform, or precipitate [23–26]. Geochemical and hydrological activity can even lead to the dissolution of bones and the formation of authigenic phosphate minerals [27, 28]. Thus, there is a “background noise” of inorganic and organic materials in the sediments that researchers who investigate lithic residues must take into consideration.

Here we establish that the challenge of contamination can be more pervasive and insidious than is generally appreciated. We illustrate this with case studies from two Middle Palaeolithic (MP) cave sites: Lusakert Cave 1 in Armenia and Crvena Stijena in Montenegro. Artifacts from both sites served as test subjects in our development of more scientific (i.e., reproducible and reliable) residue analysis methods, given that the conventional means of residue analysis with VLM alone are inadequate [9]. While working to image micro-residues using SEM and to chemically characterize them using μFTIR, we stumbled upon unexpected results. Below we present our findings in two parts. First, we show that lithic artifacts from Lusakert Cave 1 were handled carefully, following special protocols in both the field and laboratory, yet were nevertheless tainted by a modern-day contaminant from an unexpected source: a chemical inside the ubiquitous zip-top plastic sample bag. Second, we demonstrate that, when non-artifact (i.e., geofact) “controls” are studied alongside artifacts from Crvena Stijena, such comparisons reveal that organic residues occur throughout the associated sediments. Consequently, any interpretation of residues on artifact surfaces must control for this “background” presence of organic matter. Subsequently, we offer protocols for reducing contamination and, consequently, increasing the reliability of lithic micro-residue studies. Ultimately, we argue that researchers working in the field of residue studies must begin with the null hypothesis that any observed miniscule organic substances reflect contamination, either ancient or modern, and systematically proceed by ruling out all potential sources of contamination before claiming that the microscopic residues directly attest to past use activities of the artifacts.

## Part 1: Investigating modern contamination

In the following sections, we document modern contamination discovered on a set of obsidian artifacts collected specifically for residue analysis during excavations at the MP site of Lusakert Cave 1. Despite following anti-contamination protocols in both the field and lab, the artifacts were nevertheless tainted by an unexpected source: a substance commonly used inside plastic bags by their manufacturers to make them easier to open. All field and laboratory work described in these sections were conducted under the approval of the Institute for Archaeology and Ethnography, National Academy of Sciences, Republic of Armenia.

### The site: Lusakert Cave 1

**Fig 1:**
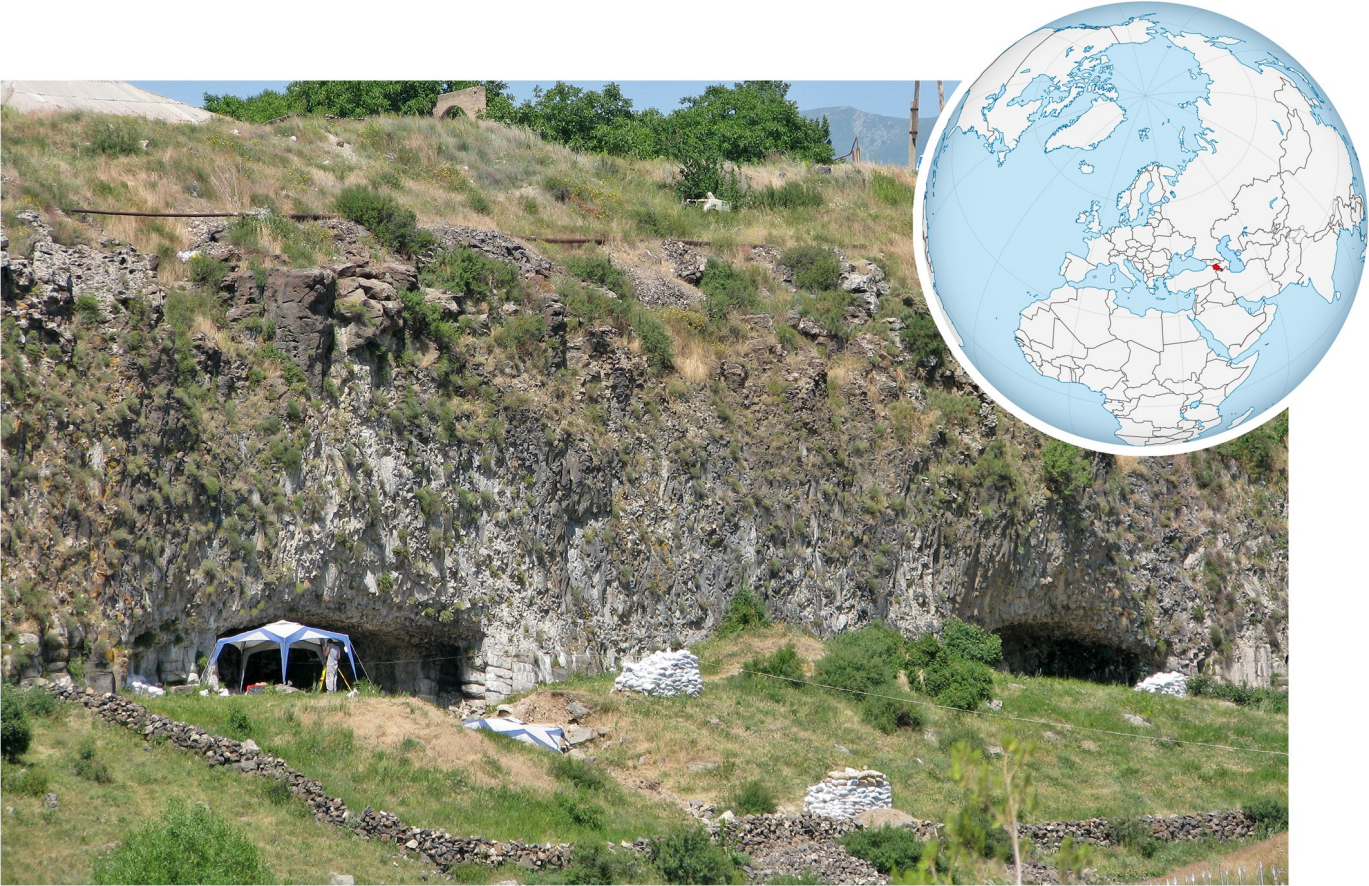
Lusakert Cave 1 (left) and Cave 2 (right). These two caves formed in a basalt cliff along a former meander of the Hrazdan River in central Armenia. Photograph taken by the first author (EF). World orthographic projection shared via Wikimedia Commons with a Creative Commons license.

**Fig 2:**
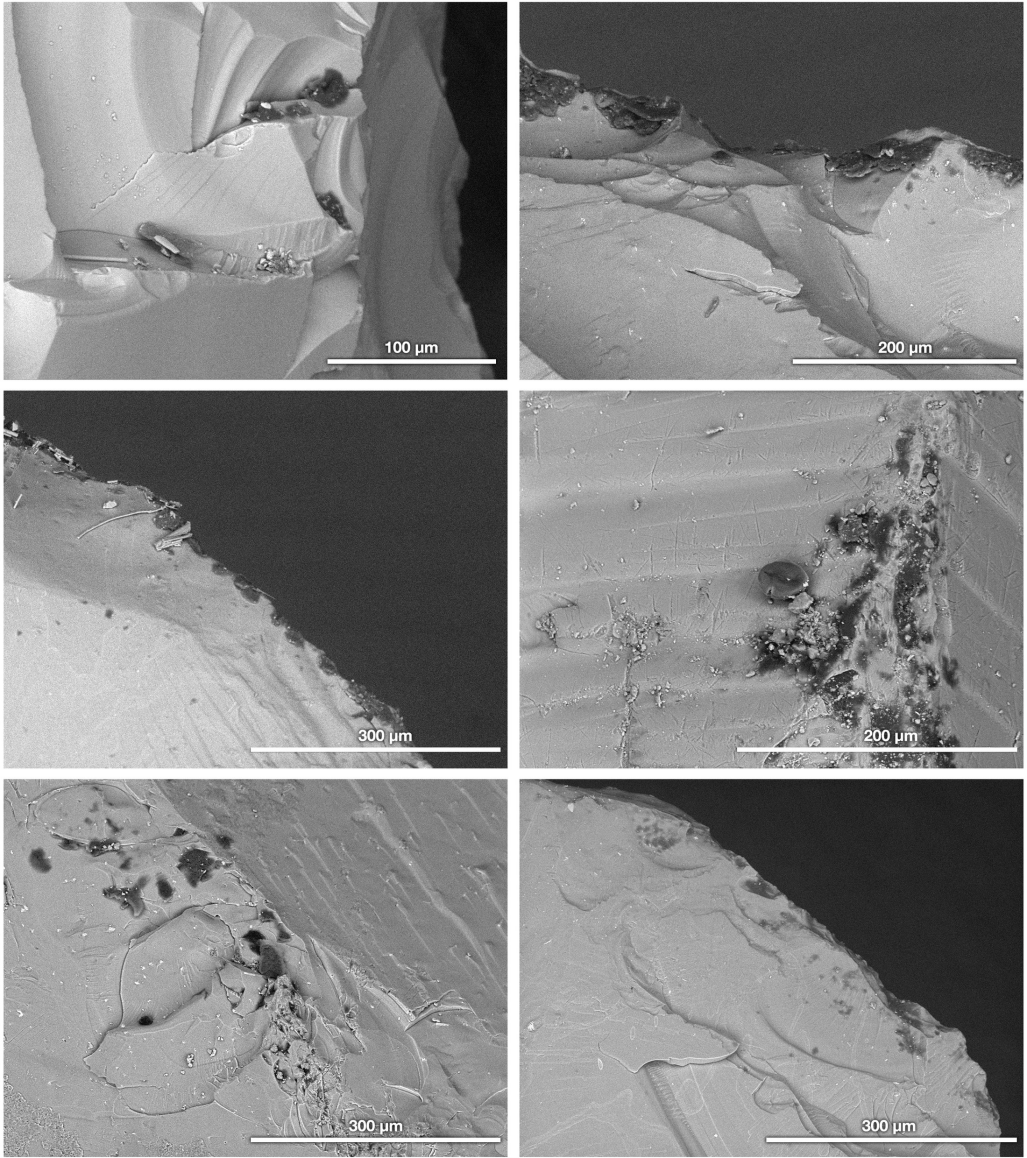
SEM-BSE images. Examples of organic residues (dark patches) on LKT1 lithic artifact surfaces. Images by the joint main authors (EF and GM).

Situated in central Armenian Highlands (Armenia), Lusakert Cave 1 (LKT1; Fig 1; 40.3717° N, 44.5972° E) formed in a 200-ka [29] basalt cliff along a cut-off meander of the Hrazdan River, which flowed past the cave entrance when it was occupied by MP hominins ca. 60–35 ka [30, 31]. After excavations in the 1970s and 1980s, which yielded a small faunal assemblage and more than 200,000 obsidian artifacts [32, 33], excavations by the Hrazdan Gorge Palaeolithic Project were conducted from 2007 to 2011 as a collaborative venture between the Institute of Archaeology and Ethnography (National Academy of Science, Armenia), University of Connecticut (USA), and University of Winchester (UK) [30, 34]. Beneath the uppermost stratum, the site’s stratified deposits contain in situ faunal remains and lithic artifacts, including 14,000 obsidian artifacts (>25 mm) from 11.9 m^3^ of sediment (smaller debitage is even more abundant), as well as intact hearth features and chemical evidence for pyrotechnology (i.e., on-site fire production [35]). Its lithic assemblage is Levallois (both flake and blade) with plain and facetted platforms, a moderate frequency of retouched tools (e.g., side scrapers, end scrapers, burins [30, 34]), cores on flakes, and kombewa pieces. During the 2011 excavations, a set of obsidian artifacts, especially those exhibiting the characteristics of Levallois flakes or blades, were collected specifically for residue analysis (Fig 2). As noted below, each of these artifacts was selected while still embedded in the profile. Complete techno-typological, stratigraphic, environmental, and geochronological studies for LKT1 are forthcoming.

### Artifact handling in the field

A random sample of 36 obsidian artifacts from six stratigraphic layers was selected during the 2011 LKT1 excavations to be examined for residues. All selected artifacts were identified and collected using a trowel (i.e., never touched by hand) by one of the authors (DSA), recorded in three dimensions using a Leica total station, and placed directly into a new, clean plastic sample bag, covered in adhered sediment. In addition, each sample was specially labeled as an artifact for residue analysis. While other artifacts were washed to remove sediments, cleaned to remove any carbonate concretions, labeled, re-bagged, photographed, and sometimes illustrated, all artifacts chosen for residue analysis skipped such processing, and they were not handled until reaching our laboratory at the University of Minnesota.

### Handling in the laboratory

The artifacts were never touched with bare skin (at least, not since MP hominins last used them). Instead, sterile, powder-free, disposable nitrile gloves were always worn. The artifacts were never placed directly onto laboratory or equipment benches. They were first carefully removed, using gloved hands, from the zip-top plastic bags into which they had been placed in the field along with adhering sediment. Next they were placed, individually, in a clean beaker with distilled water and ultrasonicated for one minute to dislodge the adhering sediment. After this washing procedure, the water was filtered to recover the sediment and other potential particles [36], if needed at a later point in time. Each artifact was laid out to dry onto a clean, new sheet of uncoated heavy-duty aluminum foil (i.e., not the “non-stick” foil varieties with polymer coatings that include silicone resin, curing agents, etc.). Once dry, the artifacts were individually stored in clean, new zip-top plastic bags. Before microscopic examination, dust was blown off an artifact with compressed CO_2_ gas. We did not use commonly available compressed gas dusters (a.k.a., air dusters; like those used to clean computer keyboards) as these contain fluorocarbons and hydrocarbons, such as propane and butane, that would likely contaminate the artifact surfaces.

### Microscopic methods: VLM and SEM

The LKT1 artifacts were first screened for the presence of microscopic residues with a combination of VLM and SEM. First, we inspected the artifacts following our previously published protocols [9] with a Leica MZ16A stereomicroscope, which is capable of total magnifications from 10× to 150× using a 1.0× planapochromatic objective lens and is equipped with a Leica IC 3D camera for viewing digital images. Each artifact was placed on a new, clean sheet of weighing paper to avoid cross-contamination from the microscope stage. Most of these observations were conducted at standard magnifications of either 50× or 115×. It should be emphasized, however, that the goal of these observations were simply to recognize the presence of material adhered to the artifact surfaces, not to make identifications of such material.

Second, the same series of artifacts was examined with a Hitachi TM-1000 SEM. This instrument can operate in a low-vacuum (sometimes called “atmospheric") mode so that it was unnecessary to apply an electrically conductive coating (e.g., evaporated gold or carbon). Each artifact was placed on a new aluminum SEM stub using a standard double-sided carbon tab. The artifacts’ surfaces were examined at magnifications between 100× and 1000× using the SEM’s high-sensitivity, solid-state backscattered electron (BSE) detector (i.e., a dedicated BSE semiconductor detector rather than an Everhart-Thornley scintillator system used as a BSE detector). In BSE images, any organic residues invariably stand out as dark patches on the artifacts’ surfaces (Fig 2), whereas adhered inorganic sediments or minerals appear brighter due to their higher average atomic number (a well-documented phenomenon that is known as “Z contrast” [37]). The SEM is outfitted with an energy-dispersive X-ray spectrometry (EDS) system, but as is standard for this model, the thermoelectrically cooled (i.e., liquid N_2_-free) detector is covered by a Be window that inhibited the characteristic X-rays from elements lighter than Na (Z = 11). Consequently, the spectra included none of the primary elements (C, O, N) for organic compounds; however, even with an ultra-thin EDS window that allows these light elements to be detected, the data are too imprecise to differentiate a majority of organic compounds (especially without the ability to measure H). Additionally, no secondary electron imaging was possible because such detectors cannot function under low-vacuum conditions with uncoated specimens given the effects on low-energy electrons.

The artifacts’ edges were prioritized for examination using both VLM and SEM, and the locations of observed organic substances were recorded. Ultimately, those artifacts that we observed to have the most abundant adhered organics were prioritized for chemical characterization using μFTIR.

### FTIR methods: Instrument and protocols

As with our previously published studies [11, 12], we acquired the μFTIR spectra using a Nicolet Continμum FTIR microscope paired with a Nicolet iS50 FTIR bench, which is housed in and administered by the University of Minnesota’s Characterization Facility. Its highly sensitive mercury cadmium telluride (MCT) detector has a spectral range of 4000–650 cm^-1^, and we operated the system in reflectance mode with a spectral resolution of 4 cm^-1^. To minimize electronic noise, the detector was cooled with liquid N_2_, and dry air was pumped through the system to reduce the effects of ambient moisture.

The advantage of μFTIR is that this technique channels the ability to provide molecular analyses (like that of conventional FTIR) through a high-powered VLM microscope that enables the infrared laser beam to be aimed at a small spot of interest. Consequently, using our prior observations from VLM and SEM and our notes regarding residue locations on each artifact, we were able to re-find these residues under the μFTIR microscope, digitally record a VLM image, and collect a μFTIR spectrum.

Windows-based OMNIC software was used for data collection and analysis. A 15× objective lens was used. The stage was cleaned with isopropyl alcohol between each artifact. The aperture dimensions were variable from 50 to 150 μm, vertically and horizontally, to select a specific area for measurement. Given the minuscule sizes of the residues, the number of FTIR measurement scans was increased from a typical range (16–64 scans) to much higher numbers (500–8000) in order to obtain the clearest possible spectra. When the aperture is reduced to 50 × 50 μm, the analysis area is about one-tenth of the 150 × 150 μm maximum area, and there is a corresponding drop in the infrared signal’s strength, necessitating much longer spectral acquisition times. Given the considerable measurement times involved (and, thus, greater instrument use fee per artifact), one third of the full sample (i.e., 12 of the 36 artifacts) with the most residues observed via VLM and SEM were the focus of our μFTIR investigations.

### Potential sources of contamination

In addition to reducing potential contaminants, we collected μFTIR spectra for substances that we considered most likely to adulterate the artifacts. Regarding contaminants, Hayes and Rots [16] observed “abundant cellulose, skin flakes, and synthetic fibers… on most tools” due to a combination of handling and airborne exposure after their ultrasonic cleaning. Skin flakes, in particular, were menacing, and they discovered that, “even when specific protocols are put in place to limit contamination, … the presence of skin flakes increased with each successive stage of analysis” (3097). Similarly, Pedergnana et al. [21] believed that skin flakes might be the most frequent contaminant, transferred onto artifact surfaces via handling during excavation and/or examination. Given that humans tend to shed skin flakes at a rate of 30–90 mg/hour [38], the signature of such contamination is worth considering.

**Fig 3:**
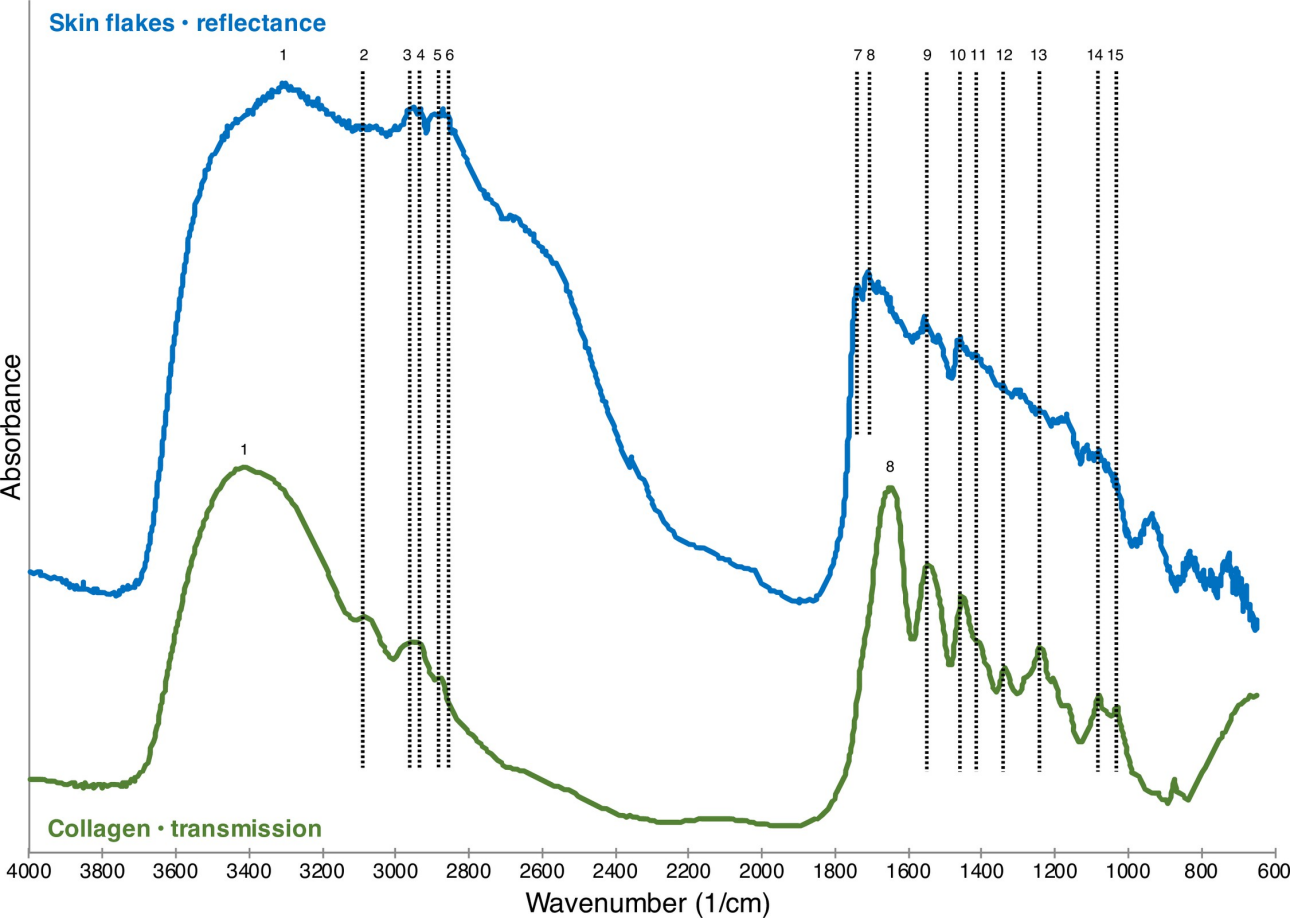
FTIR reflectance spectra for skin flakes. A μFTIR spectrum of fresh human skin flakes compared to the transmission spectrum for collagen from the reference library of Kimmel Center for Archaeological Science, Weizmann Institute of Science. The spectra have been vertically offset for clarity. We propose the following peak identifications based on our earlier publications (see Monnier et al. [12] for further details): 1. O-H stretching, amide A; 2. amide B; 3. CH_3_ asymmetric stretching; 4. CH_2_ asymmetric stretching; 5. CH_3_ symmetric stretching; 6. CH_2_ symmetric stretching; 7. C = O stretching; 8. C = C stretching, amide I; 9. amide II; 10–12. CH_x_ bending; 13. amide III; and 14 and 15. C-N, C-O, C-C stretching.

We expected that the compressed CO_2_ would remove most loose particles and stray fibers that merely settled on the artifacts’ surfaces from an airborne source. Skin flakes, however, did seem to be the most likely contaminant outside of a clean lab. To this end, we examined and collected μFTIR spectra from skin flakes (donated by one of the authors). Fig 3 shows typical μFTIR reflectance spectra for the human skin flakes in question compared to a transmission spectrum for collagen (as a KBr pellet) from the FTIR library of the Kimmel Center for Archaeological Science, Weizmann Institute of Science. As expected, the spectra were consistent with the skin flakes principally being collagen and/or keratin, given that these two proteins are structurally similar and, therefore, exhibit virtually identical FTIR spectra.

We found that the spectra for human skin flakes are similar to those we acquired for specimens of deer skin in our prior publication on experimental animal tissue residues [12]. The caption of Fig 3 lists our proposed absorption band identifications for the human skin flakes. A number of peaks remain unattributed to particular molecular vibrations but are still replicated in both the skin flakes and the collagen standard. Also note that small shifts between the reflectance and transmission spectra are to be expected given our previous work [11, 12] and might correspond to various phenomena: relative masses of the vibrating molecule, temperature, ordering and strains internal to the specimens, and so on. Additionally, differences between the skin flake spectra and collagen standard may reflect the presence of other compounds in skin, such as lipids, water, and proteoglycans [12]. Ultimately, however, any fresh human skin flakes that had recently contaminated the artifacts would be recognizable through a spectral comparison to reference materials such as those documented in our past work [12].

### Findings and interpretations

**Fig 4:**
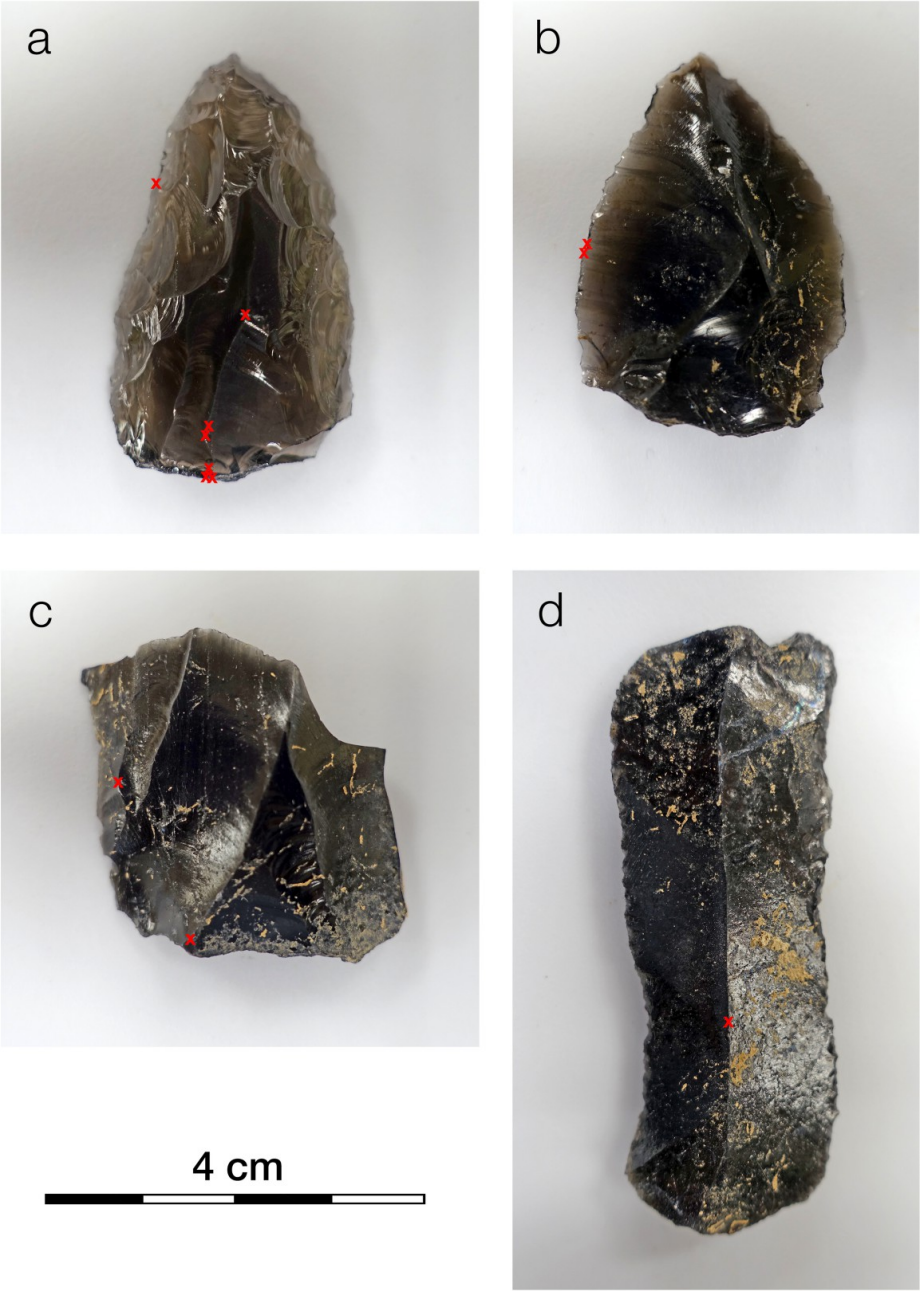
Four of the twelve LKT1 artifacts examined with μFTIR. Locations (the red X symbols) on a: G04-1026, b: H05-699, c: H05-477, and d: I05-1051 with the presence of organic residues identified by VLM and SEM. Residues on these artifacts are discussed in the text. Photographs by the first author (EF).

Organic residues were observed using VLM and SEM along the LKT1 artifacts’ edges, where it is typically thought that use-related residues should be located, and along their proximal edges, which could, at least in theory, have served as the potential base for hafting to a wooden shaft (Fig 4). The small sizes of many of the observed residues (often ≤ 60 μm in maximum dimension) made it challenging to obtain clear μFTIR reflectance spectra. The aperture size (normally 150 × 150 μm) was reduced to capture the IR signal from the residue, rather than the surrounding stone (see [12] for a discussion on the effect of a stone substrate on the IR signal of an organic residue). Reducing the aperture to ≤ 60 μm meant that the signal was very weak. Consequently, collection times had to be greatly extended (i.e., several thousand scans that often took more than an hour or two to acquire). This approach yielded interpretable spectra, although some were affected by specular reflection, which results in derivative bands [12].

**Fig 5:**
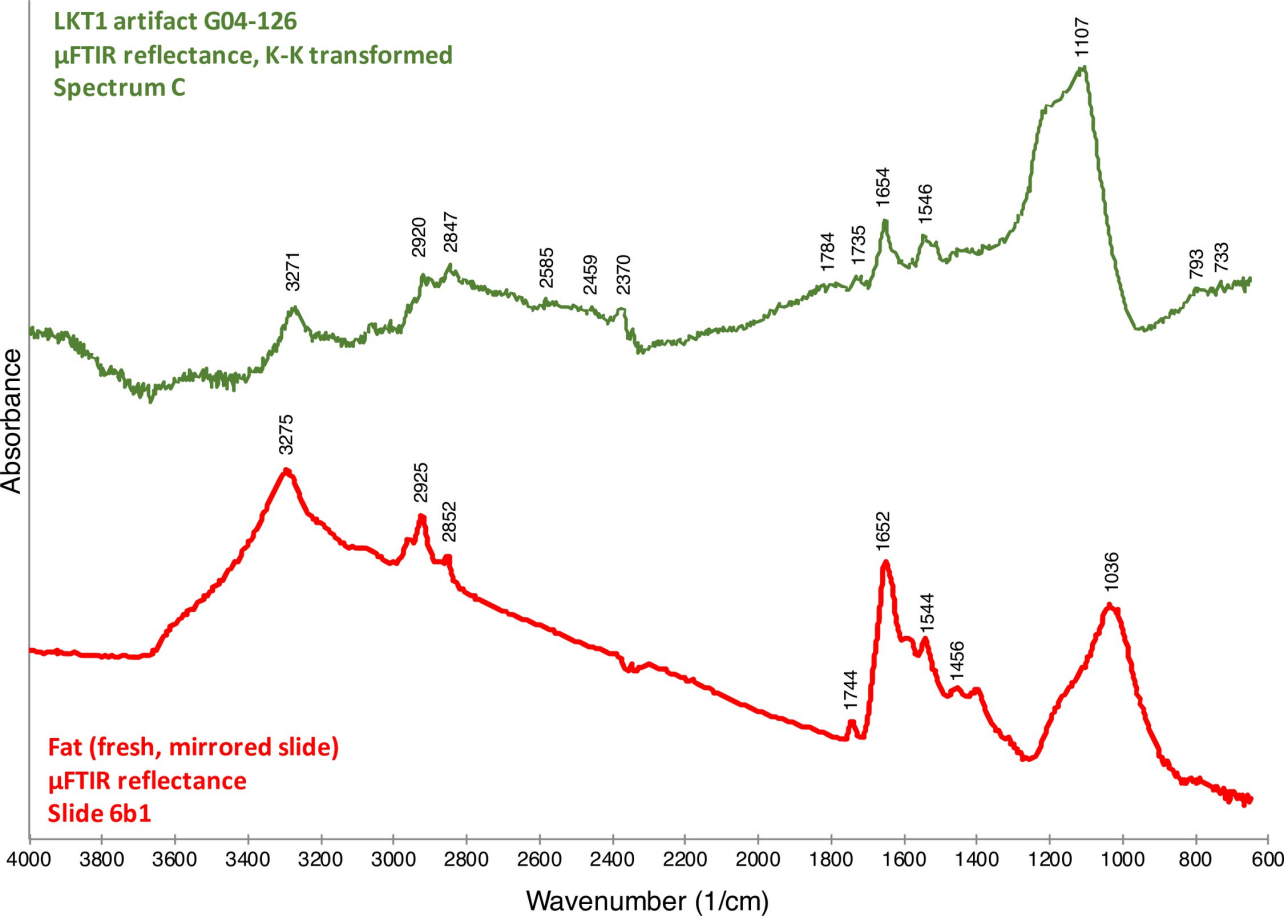
μFTIR reflectance spectrum for a micro-residue on LKT1 artifact G04-1026 (Fig 4A). Comparison between a micro-residue μFTIR reflectance spectrum for LKT1 artifact G04-126 (green) and the μFTIR reflectance spectra for fat (red)–see Monnier et al. [12] for peak attributions. These spectra have been vertically offset for clarity.

**Fig 6:**
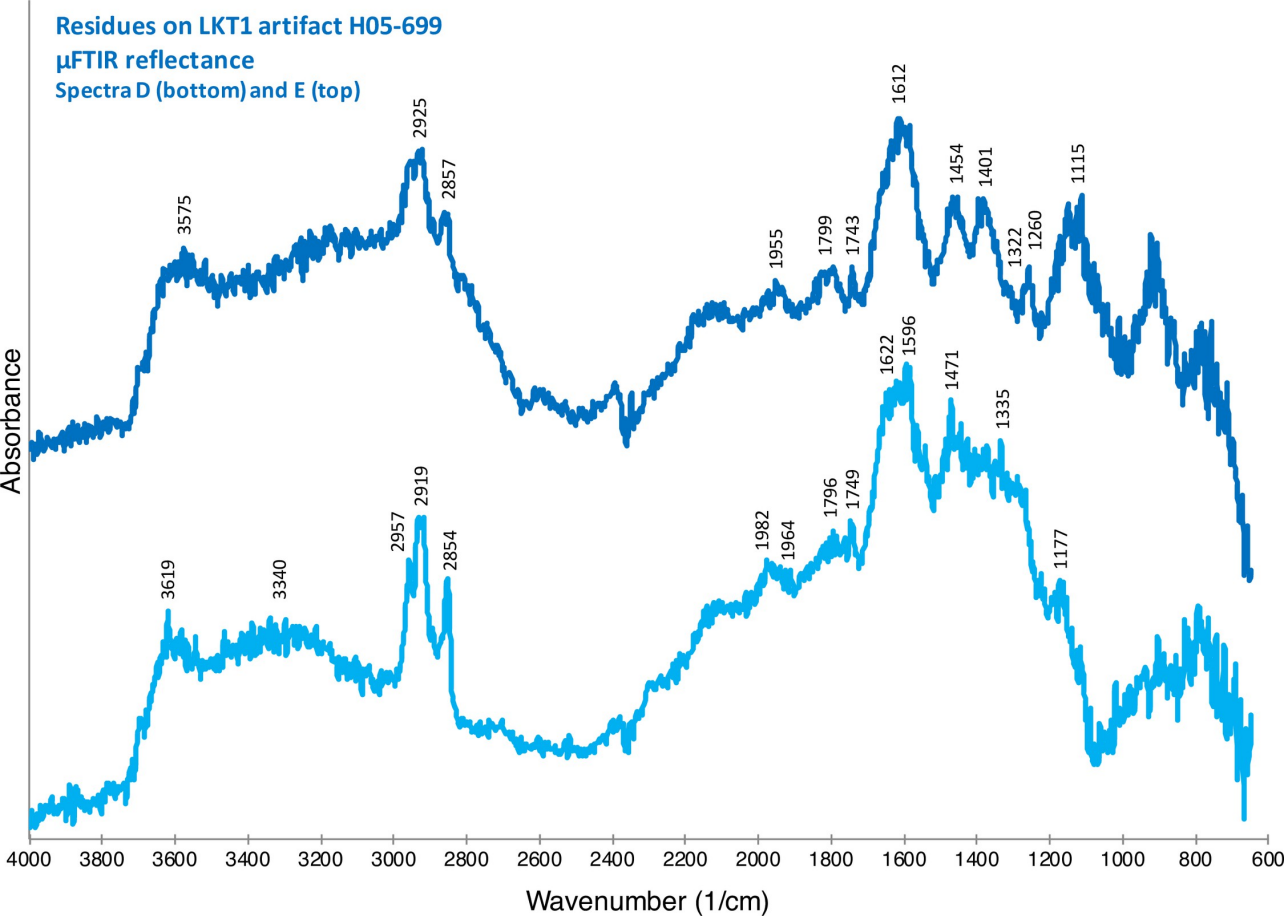
μFTIR reflectance spectra for micro-residues on LKT1 artifact H05-699 (Fig 4B). These spectra have been vertically offset for clarity. We propose the following peak identifications: 3619/3575: probable OH stretching; 3500–2700: complex band possibly reflecting a mixture of carboxylic acids, amino acids and their salts, amides, etc.; 2957: CH_3_ asymmetric stretching; 2919: CH_2_ asymmetric; 2854: CH_2_ symmetric; 2120 and 1950: unknown; 1749/1743: probable C = O stretching (from carboxylic acids, amino acids, etc.); 1700–1530: multiple species, including water OH bending, NH bending, amide I and amide II in proteins, amino acids and amides, C(O)O asymmetric stretching in carboxylic acid salts; 1500–1400: likely CH_x_ bending; and 1259 and 1150: probable C-O, C-N and C-C stretching. The locations of these residues are indicated by the two red X symbols in Fig 4B.

The spectra confirmed the organic nature of the residues, and to interpret them, we turned to the reflectance μFTIR standards that we had previously developed [11, 12] and to the literature. Fig 5 illustrates the spectrum from a micro-residue on G04-1026 (Fig 4A), which appears to be composed of animal fats (in particular, triglycerides). Notice that the Kramers-Kronig (K-K) transform has been applied to the spectrum in this instance because specular reflection produced derivative bands (see [12]). The large and broad peak present between 1300 and 1000 cm^-1^ is due to a *reststrahlen* band that arises due to specular reflection from the obsidian substrate. The rest of this spectrum, though, exhibits peaks largely consistent with our reflectance standard for animal fat [12]. Therefore, our current identification, albeit provisional, for this micro-residue on G04-1026 is animal fat. A different artifact–a Levallois point, H05-699 (Fig 4B)–exhibits a chemically distinct residue on its edge. Fig 6 shows two spectra for this residue. The spectra are difficult to interpret precisely but, in general, indicate a complicated mixture of organic compounds including carboxylic acids, amino acids and their salts, and amides. Our interpretation of these spectra–again provisional at present–is that they show a presence of organic compounds likely related to protein degradation products such as amino acids, amino acid salts, amides, etc.

The other spectra, however, exhibited strong peaks around 3400 and 3200 cm^-1^. This indicated a functional group that had not been identified during the development of our residue standards, including those for animal fats and proteins. Further research revealed that these peaks, along with the one at 1650 cm^-1^, are consistent with primary amides, which have the formula of RC (= O)NH_2_. The peaks at 3400 and 3200 cm^-1^ are attributed to NH_2_ asymmetric and symmetric vibrations, respectively, and the 1650 cm^-1^ peak is the carbonyl (C = O) group. Proteins have secondary and tertiary amides, but not primary amides, the presence of which in the spectra needed to be explained. Thus, the presence of the primary amide remained a mystery for some time. Finally, while further investigating the spectral data, which indicated that these substances must contain long aliphatic chains (because the observed CH_2_ peaks are so strong), we discovered a clear match to the reference spectrum for a particular chemical compound: stearamide, which is also known as octadecanamide and has a general formula of C_18_H_37_NO.

**Fig 7:**
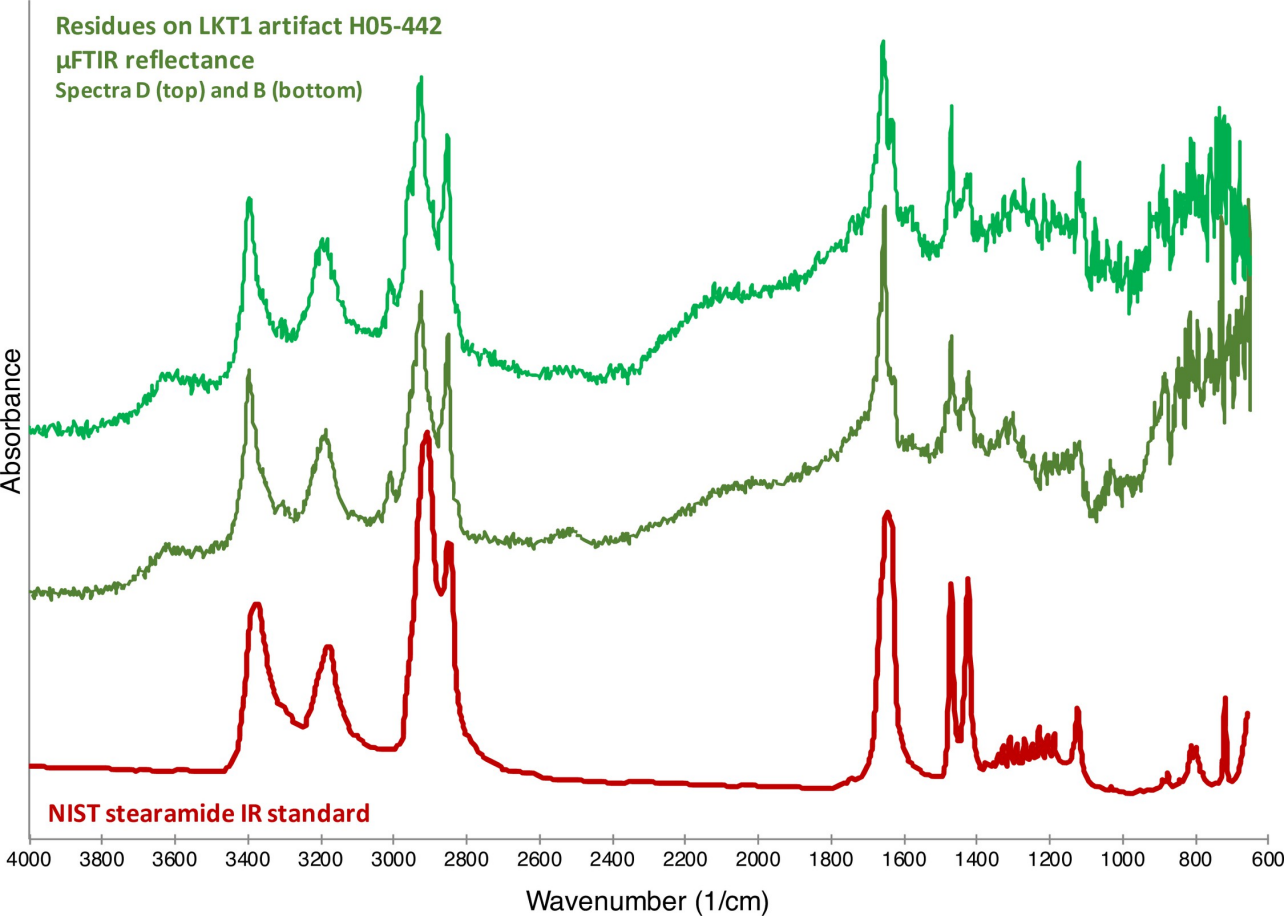
Artifact residues vs stearamide. Comparison between LKT1 artifact H05-442 micro-residues μFTIR reflectance spectra (green) and the spectrum for stearamide from the NIST FTIR library (red). These spectra have been vertically offset for clarity.

**Fig 8:**
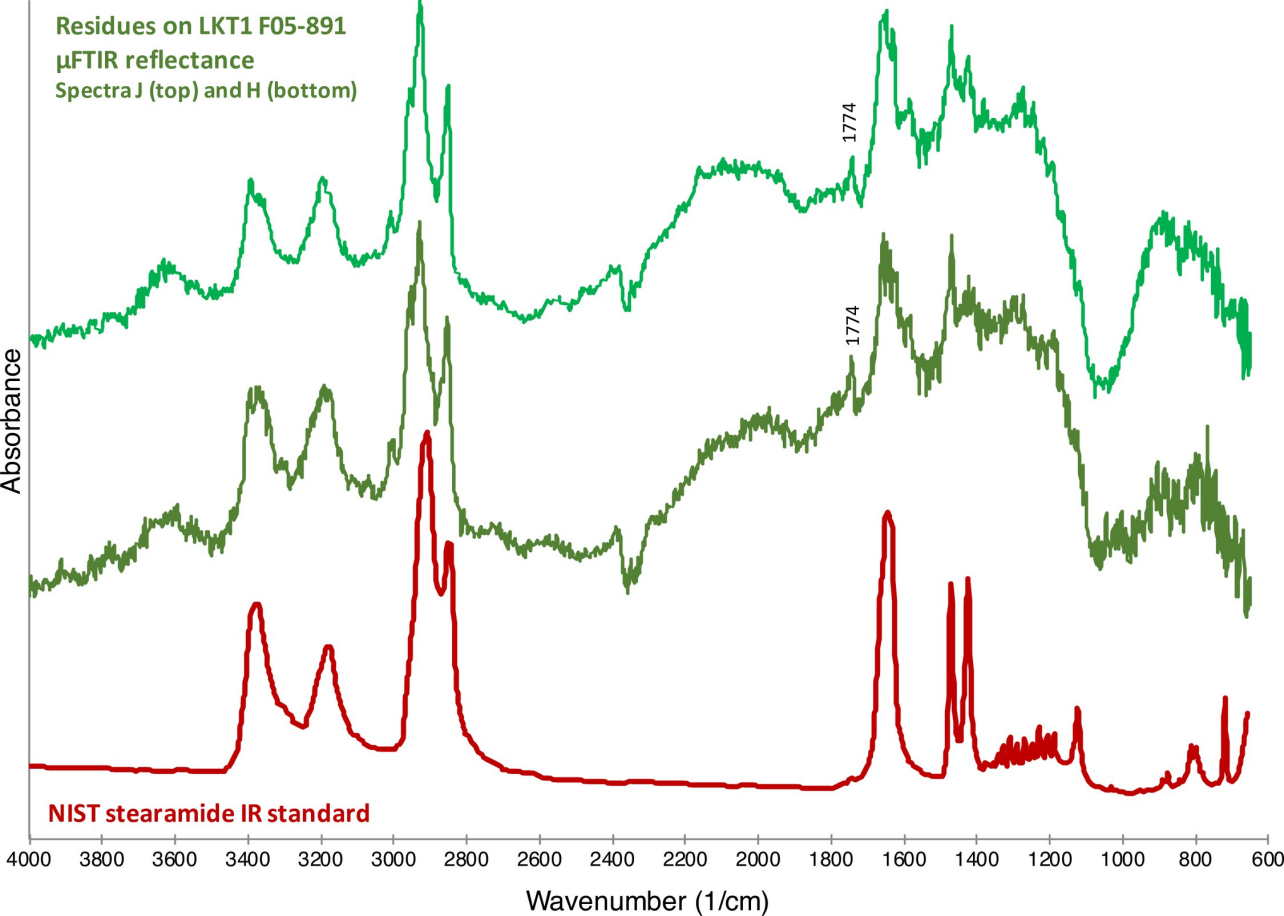
Artifact residues vs stearamide. Comparison between LKT1 artifact F05-891 micro-residues μFTIR reflectance spectra (green) and the spectrum for stearamide from the NIST FTIR library (red). These spectra have been vertically offset for clarity. The peak at 1744 cm^-1^ might indicate the presence of fatty acids in addition to the stearamide.

**Fig 9:**
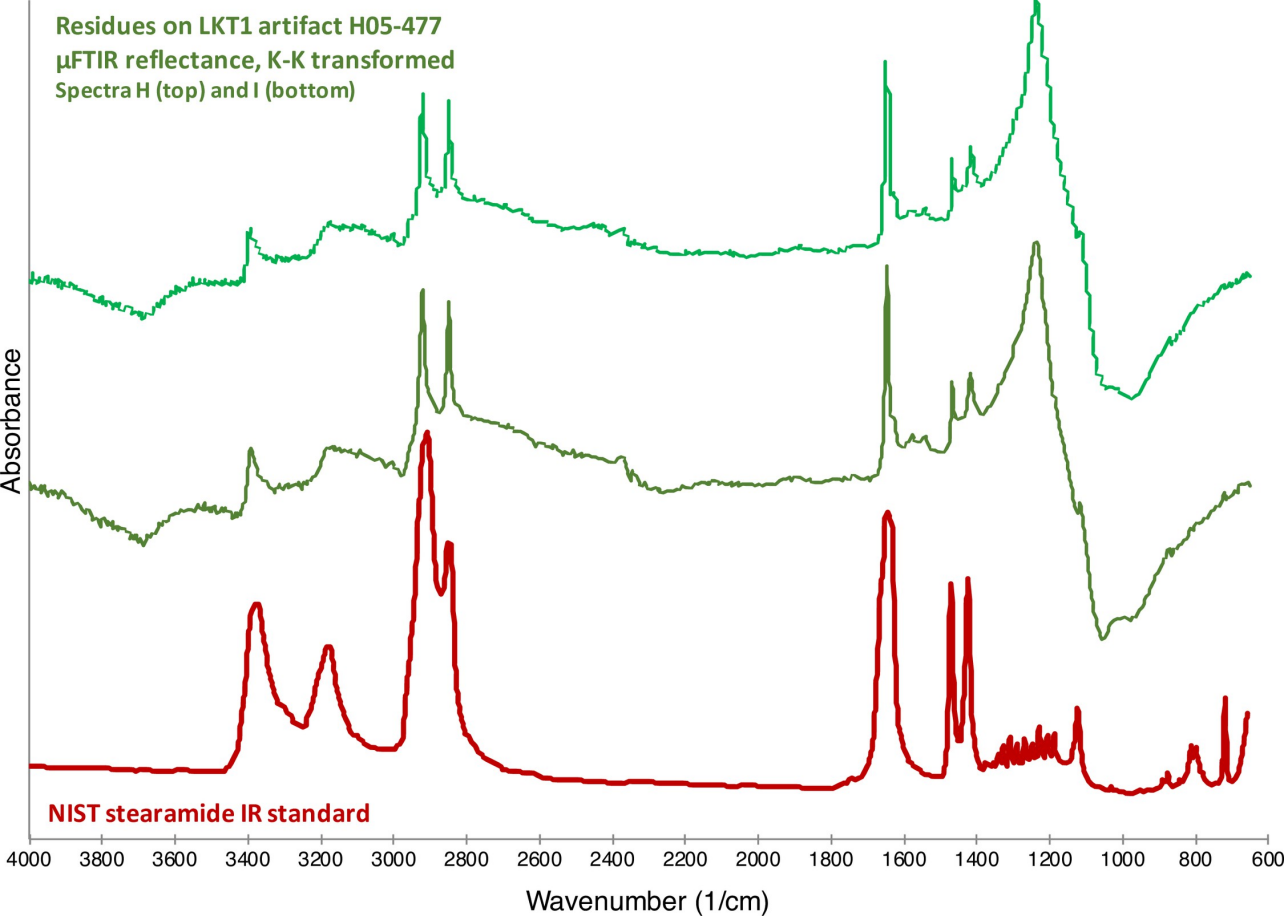
Artifact residues vs stearamide. Comparison between LKT1 artifact H05-477 micro-residues μFTIR reflectance spectra with the Kramers-Kronig transform applied (green) and the spectrum for stearamide from the NIST FTIR library (red). These spectra have been vertically offset for clarity.

As shown in Fig 7, there is little doubt that that many of the observed organic substances on the 12 analyzed LKT1 artifacts are actually composed of stearamide. The residues in Fig 8 are composed of stearamide together, perhaps, with some fatty acids, as indicated by the peak present at 1744 cm^-1^ (see the spectrum for the animal fat in Fig 5). The Fig 9 spectra both exhibit specular reflection, requiring the K-K transform; however, once applied, it can be observed that the spectra contain peaks consistent with the National Institute for Standards and Technology (NIST) standard for stearamide.

Unfortunately, stearamide is a modern contaminant. In particular, it is an industrial product that is commonly used as a release agent [39–45], especially as a lubricant to reduce the surface friction and static between plastic sheets [46], due to its long-studied properties as a coating [47–49]. Indeed, these compounds are frequently applied to the interior surfaces of plastic zip-top bags in order to make them easier to open by preventing the opposite sides from sticking together [50]. Importantly, it is known by specialists in other fields, such as analysts who conduct laboratory testing of crime scene evidence, that stearamide is a source of “transferable contamination” from zip-top bags [51]. Conservation specialists are similarly aware of the potential for contamination by stearamide. For example, the Conservation and Art Materials Encyclopedia Online (CAMEO) database [50], which is hosted by the Museum of Fine Arts Boston and was developed by its curatorial staff, clearly states:

[It is a] colorless, waxy compound. Stearamide is commonly used as a release agent on the surface of plastic bags (e.g., Ziploc®) to prevent the sides from sticking together. Small amounts of stearamide can readily transfer to the surface of any material placed inside the bags.

Thus, we have every reason to believe that the stearamide is a contaminant from inside one or more of the sample bags in which each artifact was stored. Stearamide is not water-soluble [52], so it would not be removed by a simple wash or an ultrasonic bath in water. It is only mildly soluble in alcohol, so even a wash with isopropyl alcohol, for example, could not remove it. The stearamide residues were typically found inside small flake scars and recesses (i.e., concavities) along the artifacts’ sharpest edges, like those used for cutting, and on flake ridges, given that those spots were most probable to scrape along the bag interiors and accumulate stearamide. Smoother artifacts, in contrast, exhibited fewer residues.

This outcome leads to four troubling implications worthy of attention:

1. It would have been easy for us to accept our *initial* identification of the residues as fats. Only our own endeavors to prove ourselves wrong led to finding what we now maintain is the correct identification of the adhered substance: stearamide.
2. There are “invisible” contaminants all around us. We saw no mention of stearamide as an ingredient of the zip-top sample bags. The only material that is listed in the online catalog is polyethylene plastic. Therefore, the burden lies on ourselves to consider and identify all potential sources of contamination in artifact residue studies.
3. The use of zip-top bags is ubiquitous in both archaeological research in general and lithic residue studies in particular. It is now clear that, without some additional barrier between the artifacts and the bags’ interiors, the pervasive use of stearamide as a release agent can be actually an effective means of *adding* modern organic contaminants to artifacts.
4. Use-wear analysis has been used as a means to corroborate or elaborate interpretations from residue analysis (e.g., [19, 20]), presuming that these supposedly independent lines of investigation should increase confidence in the interpretations of artifacts’ uses. If, as observed, some forms of modern contamination are more likely to accumulate in any flake scars or recesses, like those due to retouch or damage to working edges, there is an increased danger of misinterpreting such a contaminant as a use-related residue. That is, there is a potential for use-wear studies to erroneously reinforce the interpretation of a contaminant as a use-related residue due to this phenomenon.

**Fig 10:**
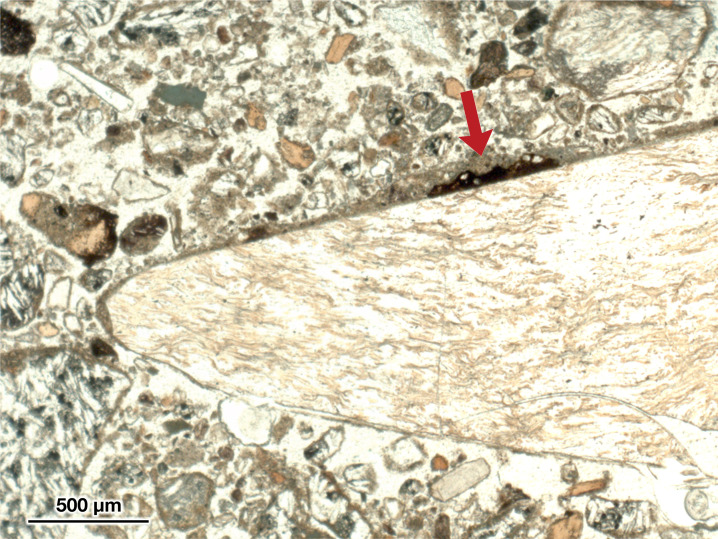
A thin section of LKT1 Unit 5 sediments. Viewed in plane polarized light, microphotograph of a sediment thin section from Unit 5 shows an obsidian flake (center) with an adhered residue (as indicated by the red arrow). Micrograph by one of the authors (CM).

We should emphasize that it is not the case that we believe there to be absolutely no ancient residues on artifacts from LKT1. Indeed, our μFTIR investigation was partially successful. The spectra for a micro-residue along the edge of artifact H05-699 indicate the presence of an as-yet-unidentified organic compound perhaps related to the degradation of proteins, whereas the residue on G05-1026 appears to be some type of animal fat (or, at least, principally triglyceride). Additionally, Fig 10 shows a petrographic thin section of LKT1 sediments from Unit 5, viewed in plane polarized light, from micromorphological investigations inside the cave. This micrograph highlights an instance of a suspected residue adhered to the surface of a small obsidian artifact, maybe a resharpening flake or perhaps either shatter or shaping debris. Unfortunately, petrographic thin sections that have been prepared with a cover slip, as this one has been, are not suitable for μFTIR (or SEM) analysis. Nevertheless, this example serves to demonstrate further that there are indeed ancient residues adhered to at least some fraction of the LKT1 artifacts; however, it also brings us to the second issue that we wish to address.

## Part 2: Investigating past contamination

It is commonly assumed that microscopic residues adhered to the surface of a stone artifacts reflect its use (i.e., functional residues), rather than a mere coincidence of its deposition. It should be emphasized, though, that not all researchers make such a presumption, recognizing that such residues may occur naturally within the artifacts’ burial environments [17, 53]. One way to consider the issue of past contamination is to examine non-artifacts, in particular geofacts (e.g., unmodified pebbles), as “controls” that have experienced precisely the same depositional conditions and post-excavation protocols. We used this approach for artifacts recovered from Crvena Stijena in western Montenegro. All field and laboratory work described in the following sections were conducted under the approval of the Ministry of Culture of Montenegro.

### The site: Crvena Stijena

**Fig 11:**
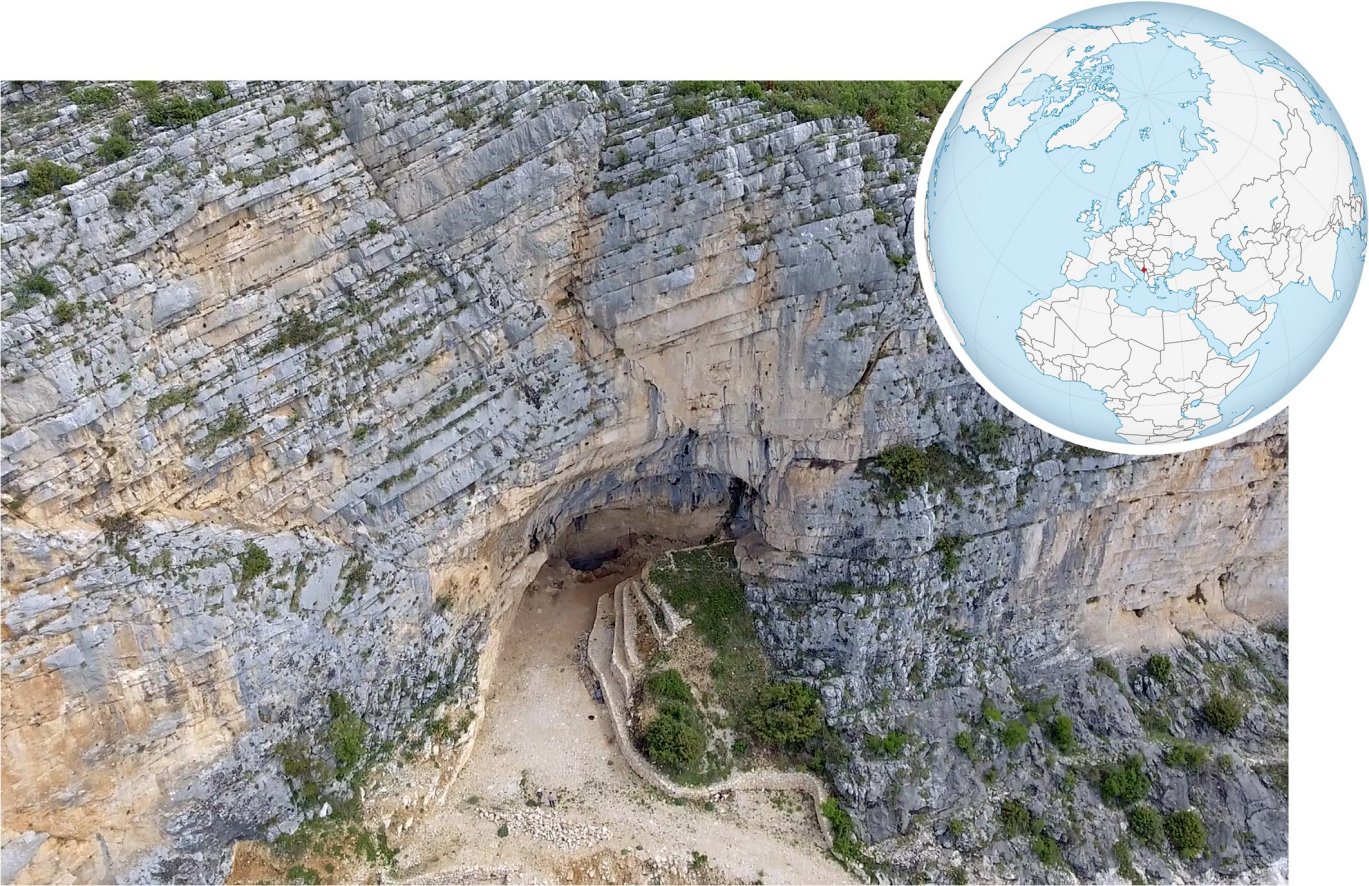
The rockshelter of Crvena Stijena. The site exists in a cliff that overlooks the Trebišnjica River. Photograph by project members Colin McFadden and Samantha Porter. World orthographic projection shared via Wikimedia Commons with a Creative Commons license.

Near the border of Montenegro with Bosnia and Herzegovina, Crvena Stijena (Fig 11; 42.7790°N, 18.4816°E) is a rock shelter along a limestone cliff that overlooks Lake Bileća, a reservoir formed by a hydroelectric dam on the Trebišnjica River. Its deep stratigraphic sequence spans the MP to Bronze Age, with the MP sequence >12 m in depth and capped by a thick layer of volcanic tephra from the Campanian Ignimbrite eruption 39.9 ka [54]. Hence, the site is known as preserving one of the deepest records of MP occupation in the Balkan peninsula, including large quantities of well-preserved faunal remains, deeply stratified hearth features, and lithic sequences that have made it a type site for the region [55–59].

Excavations during the 1950s and 1960s removed extensive volumes of material and unearthed more than 15,000 artifacts of various types [56, 59]. Excavations did not resume until the mid-2000s [60], and they continue today under an international collaboration led by the University of Minnesota and the National Museum of Montenegro [61, 62]. The ongoing excavations are focused on the upper portion of the MP sequence inside the rock shelter (Basler’s layers XII–XVII, renamed as M1–M5 [63]). Within this environment, hominins made fires, butchered animals (mostly red deer [64]) and knapped a wide variety of lithic raw materials (e.g., primarily medium- to low-quality brown-grey chert but also grey-, red-, and green-hued flint and japser; chalcedony; limestone; and a number of miscellaneous and indeterminate rocks [43]) using a mostly discoidal technology, with a weak presence of Levallois flaking. They produced scrapers, denticulates, and very small cores intensively exploited to make *ad hoc* tools, producing a ‘micromousterian’ element [57, 65, 66].

In order to test the recently developed multi-analytical residue analysis protocols [11–13], as well as to gain insight into the lithic use behaviors at Crvena Stijena, artifacts are specifically collected during each excavation season for residue analysis.

### Methods: field and laboratory protocols

**Fig 12:**
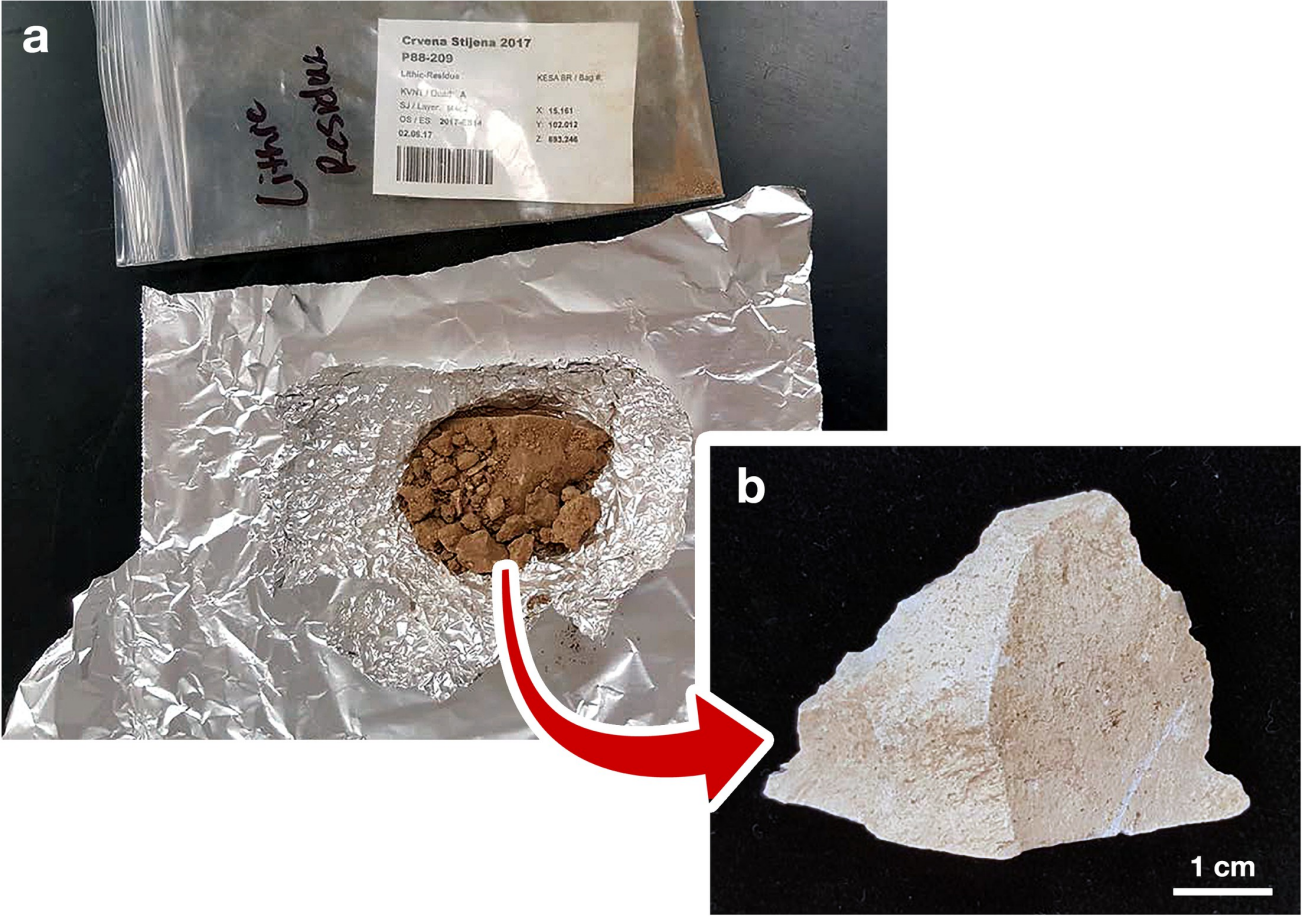
Opening an artifact packet in the laboratory. An example of (a) a sediment packet from Layer M4c2 of Crvena Stijena and (b) the lithic artifact (a proximal retouched Levallois flake) that was randomly selected for residue analysis and placed, unwashed and untouched, on the aluminum foil square.

Due to the time-consuming nature of residue analysis, it is not possible to examine the entire lithic assemblage from Crvena Stijena. Therefore, 20% of artifacts are chosen for residue analysis. In order not to privilege any specific lithic artifact types, every fifth lithic artifact that is recorded by the total station is collected for residue analysis. This strategy warns excavators when the next lithic artifact is due to be set aside for residue analysis so that it not be touched with bare hands. The excavator in question dons a new pair of nitrile gloves and, using their trowel, removes the artifact and a small amount of surrounding sediments from the deposit. Prior to 2019, the artifact and the surrounding sediments were put onto a clean sheet of uncoated aluminum foil (Fig 12). Although artifact remained in the sediment block, there was lingering concern that the aluminum sheet could abrade artifacts and, in turn, hinder any use-wear studies. When the problem of stearamide contamination was identified, a new collection protocol was developed, which consists in placing the artifact and sediment on a sheet of Tyvek conservation fabric. Additionally, excavators are instructed to include, along with the artifact and sediment, a gravel-sized, unworked clast from within a 10-cm horizontal radius of the artifact as a control. In the case that no clasts in this size category are present, excavators choose one in the pebble size class. The packet containing the artifact, sediment, and control clast (Fig 12) is then folded, and the orientation of the artifact (i.e., which surface faced upward) is marked on the packet. The packet, protected by a Tyvek barrier, is placed in a plastic zip-top bag. One corner of the packet is opened back in the field lab in Petrovići, for a period of 24 hours, to allow moisture in the sediment escape by evaporation. This was done to prevent fungal growth in moist sediments. It is then resealed and stored, unopened, until it reached the laboratory at the University of Minnesota.

In the laboratory, the packet is opened by a researcher wearing nitrile gloves. The artifact and unmodified stone are photographed and then gently rinsed with distilled water using a hand-held wash bottle. The rinsed sediments are filtered and saved. Once rinsed, the artifacts and stones are placed in separate, clean zip-top sample bags, again using a barrier around them. As with the LKT1 artifacts, the artifacts are examined using VLM (the same Leica MZ16 stereoscope) and SEM (a benchtop Hitachi instrument) to record the presence and position of organic residues. Residues were further investigated using μFTIR (the same Nicolet Continμum microscope and iS50 FTIR bench as above).

### Findings and interpretations

**Fig 13:**
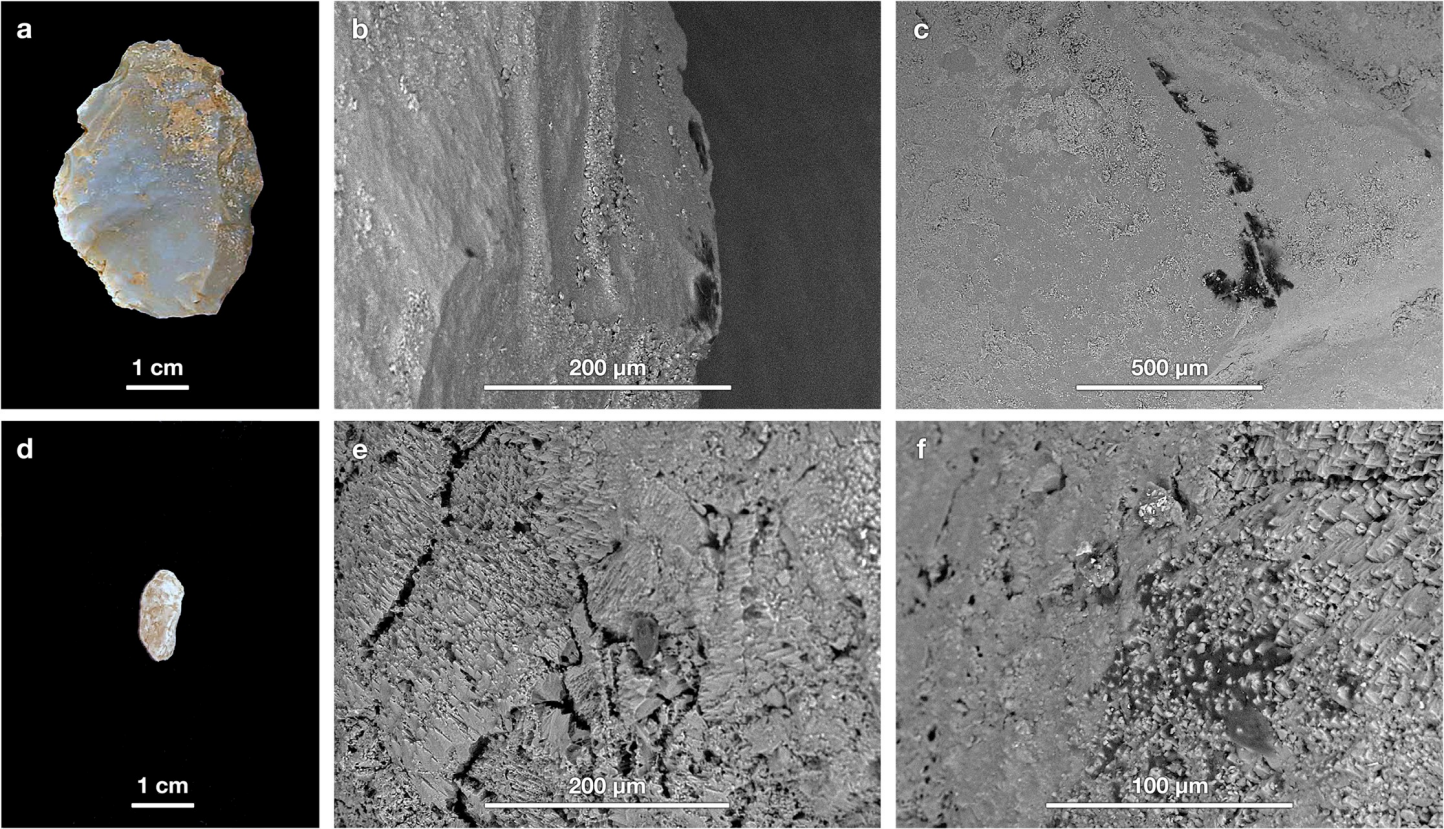
Example artifact and geofact. (a) A retouched atypical Levallois flake made of brown-grey chert and (d) an unmodified stone from sediment packet M89-9 both exhibit the presence of microscopic organic residues in SEM images (b, c, e, f).

**Fig 14:**
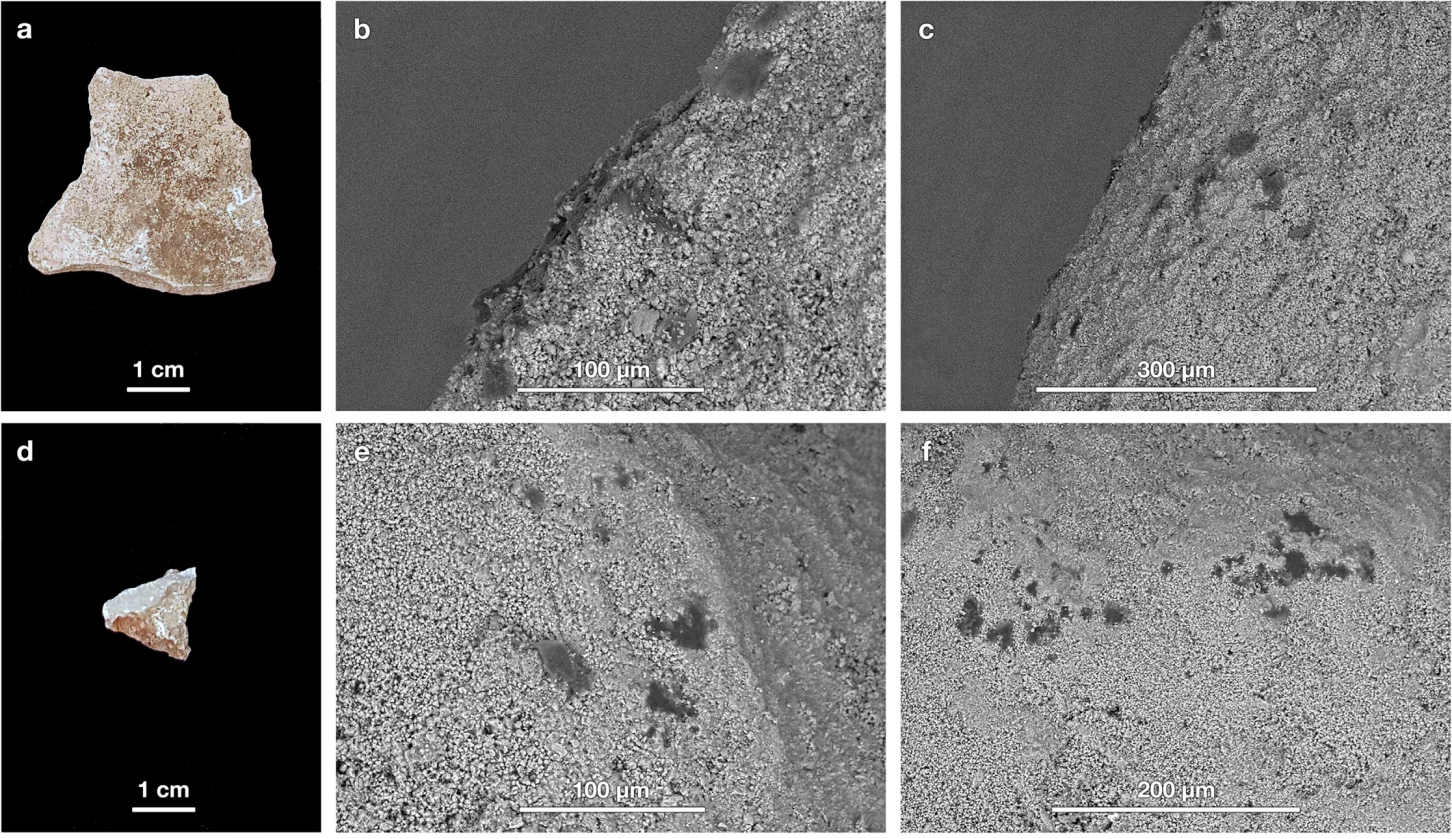
Example artifact and geofact. (a) An unretouched distal fragment of a limestone flake and (d) an unmodified stone from sediment packet P88-216 both exhibit the presence of microscopic organic residues in SEM images (b, c, e, f).

Fig 13 shows an artifact selected for residue analysis (M89-9) and the control pebble collected along with it. After the washing procedure described above, the artifact, made of the predominant brown-grey chert, was revealed to be a Levallois flake with irregular retouch along one of its edges (Fig 13A). SEM imaging reveals the presence of organic residues along the edges (see Fig 13B for an example) and on several flake ridges (as in Fig 13C). The issue is whether these organic residues are a result of either use or contamination. Checking for the presence of organic residues on the control pebble (Fig 13D) yields important information. Given that pebble is not an artifact, any residues observed on it must represent contamination from the burial environment and/or handling. Fig 13E and 13F show that organic residues are indeed present on the pebble. Another artifact submitted to residue analysis was an unretouched distal fragment of a limestone flake (Fig 14A). SEM investigation revealed organic residues along some of its edges, as seen in Fig 14B and 14C. The control pebble examined alongside it (Fig 14D) also exhibits organic residues. In the dozen artifact-geofact pairs examined to date, there have been no instances of controls without adhered residues.

These SEM images establish that there are organic residues adhered not only to the artifacts we examined but also to geofacts recovered from the same block of sediment. Unfortunately, in this case, the organic residues were too tiny (typically ≤ 30 μm in diameter) to attempt μFTIR identification, which, with a 15× objective, can only be applied to residues ≥ 50 um in diameter. Nevertheless, the presence of organic residues on the non-artifact control samples means that an added layer of caution is needed in interpreting such residues as use-related or functional in origin when identified on artifacts. Furthermore, our results highlight that the smaller the residue, the more challenging its chemical identification and, in turn, the harder it is to deduce its likely origin. Ultimately, a higher standard must be met when organic residues are present as background contamination, as we established from the artifact-geofact pairs that we analyzed for residues from layer M4 at Crvena Stijena. Other sites or other layers from the same site might yield a different outcome, whereby there are no organic residues on the controls. In such a case, one could have greater confidence that residues observed on the artifacts reflect their uses in the past. Without controls, though, there is no way to establish whether or not background organic contamination is present, and thereby no way of reliably determining whether any microscopic residues identified on artifacts are use-related or simply the result of background contamination.

## Recommendations

The above findings imply that the results of any residue study that did not follow strict anti-contamination protocols are open to doubt. Rather than calling out specific studies in which we feel that the anti-contamination procedures were inadequate, ignored, or otherwise problematic, our focus here is developing recommendations for protocols to be followed in future residue studies. It is worth noting that similar calls for greater interpretive caution and new, stricter protocols have been made in closely adjacent fields, such as lipid and biomarker analysis of archaeological remains [67].

Starting in the field, we suggest wearing nitrile gloves while excavating when residue studies are anticipated. This is a simple means to avoid contact between artifacts and exposed skin to prevent the transfer of sweat, oils, sunscreen, hand lotion, etc. When a specific artifact is chosen for residue analysis, the adhering sediment should not be removed. Instead, the artifact and associated sediment (including any small stones to use as “controls”) should be wrapped in, we propose, a sheet of Tyvek conservation fabric. One similar sampling approach, worth highlighting, has recently been used to collect lithic artifacts from Liang Bua cave (Indonesia) for use-wear analysis, specifically linking polished flakes to plant processing [68, 69]: “each was collected on and often firmly encased within a surrounding pedestal of sediment. The whole pedestal was [air-dried and] then wrapped in plastic film. All the artefacts on pedestals arrived in the laboratory in an uncleaned state with sediment attached and were subsequently excavated from the pedestals” [68].

Direct contact between the artifacts and paired geofacts should be avoided to reduce modern transfers of ancient contaminants. We do not recommend aluminum foil due to its potential to abrade artifacts and, in turn, hinder any subsequent investigations aiming to identify traces of use-wear. Tyvek conservation fabric is made of high-density polyethylene fibers, and it is nonabrasive, tear-resistant, and resistant to water, mold, mildew, and dust, making it popular for the long-term storage of artwork. Thus it is a suitable choice as a barrier between archaeological samples and potentially stearamide-coated zip-top plastic bag interiors. The orientation should also be noted (e.g., which side was facing up/down) given that studies have demonstrated that residue preservation is often superior on the bottom surface [8, 70]. The Tyvek can be folded into a packet in such a way that it stays closed, or heat sealing (using a portable or handheld device) can bond the polyethylene fibers to themselves. The Tyvek packet can then be placed inside a zip-top plastic bag for protection while still preventing contamination.

Sterile nitrile gloves must also be worn at all times when handling artifacts in the lab. It should not be thought that the artifacts can be handled without gloves (e.g., during illustration or labeling) and then simply be cleaned afterward to remove oil, sweat, or other modern contaminants. After opening the Tyvek packet, the artifacts should be gently cleaned–either rinsed using a wash bottle or agitated in an ultrasonic bath for a brief period (approximately a minute, but this may vary case-by-case)–with distilled or deionized water to remove adhered sediment. Compressed CO_2_ gas must be used instead of cheap “air duster” cans, which contain abundant contaminants, to blow off any particles that settle onto the artifact surfaces. When examining an artifact, it should never come into contact with foreign substances, even if common in many laboratory settings (e.g., Sticky Tack® putty or Parafilm®, which is composed of waxes and hydrocarbon-based polymers made of olefins), or placed on surfaces that have not been sterilized. One protocol that we endorse is that of Bordes et al. [71], who use a sterile nitrile glove as the surface on which artifacts are placed under a Raman microscope. Finally, one must be familiar with not only general lab contaminants but also those specific to spaces in which one is working (e.g., Are powdered gloves often used in that room? What other sample processing occurs in that laboratory?). Following Crowther et al. [22] regarding monitoring potential starch grain contamination via airborne routes, we propose that researchers place slides with double-sided sticky tape alongside any exposed artifacts in order to remain cognizant of airborne contamination within a particular laboratory space.

Compared to macroscopic residues (e.g., bitumen traces on Levallois points from Hummal, Syria; 13), microscopic residues might be more likely to reflect contamination and, in turn, necessitate additional care in their interpretation. Microscopic contaminants–modern as well as ancient–will always pose challenges, even when minimized through the implementation of strict anti-contamination procedures. As we document, even when the last hands to touch the studied artifacts were those of MP hominins, modern and ancient contamination can lead to residues entirely unrelated to use.

## Conclusions

It would be compelling to discover residue evidence for butchery and related activities on the MP lithic artifacts from LKT1 or Crvena Stijena. The faunal remains at the sites attest that butchery activities occurred. For example, Adler et al. [30] report that the faunal assemblage of LKT1, which is primarily *Capra* sp. and *Equus* sp., is highly fragmented due to such activities. Splintered bones exhibit percussion marks (e.g., pits, conchoidal notches) and green breaks, revealing the consumption of marrow from long bones, and cut marks on the bones are much more common than carnivore gnaw marks. Similarly, the MP layers at Crvena Stijena contain abundant faunal remains, especially red deer, and given the prevalence of cut marks on bone, the site’s occupants may have been focused on processing large game for consumption [64]. Consequently, the zooarchaeological evidence for the occurrence of butchery activities at the two sites is convincing. Given that MP individuals likely used their lithic implements in a number of ways, the value of residue analysis at such sites is the potential to clarify which artifacts were used in which ways (e.g., some used for butchery, others for woodworking, yet others for both). So far, there are tantalizing clues that our μFTIR investigations have been at least partially successful at recognizing ancient residues on two artifacts from LKT1: provisional chemical traces of proteins and animal fat.

Our focus here is not to report systematic residue analyses of lithic artifacts from either LKT1 or Crvena Stijena. Rather we communicate unanticipated findings that arose during ongoing micro-residue studies of artifacts from these two sites. Through our long-term research efforts to develop more reliable and reproducible methods of documenting and characterizing lithic residues, we came across evidence of contamination, both ancient and modern, including sources that, to our knowledge, have hitherto been overlooked in the literature. Using μFTIR, we chemically identified stearamide, which is widely used as a release agent inside zip-top plastic bags, adhered to the surfaces of LKT1 artifacts carefully collected for residue analysis. For artifacts from Crvena Stijena, we implemented a new protocol–the first of its kind, to the best of our knowledge–involving simultaneous studies of artifacts and non-artifact controls from the very same contexts. SEM observations revealed the presence of organic “residues” on such controls, implying that, only if residues on the artifacts are distinct, can they be interpreted as possibly use-related rather than a product of background organic matter within the burial environment.

In summary, both modern contaminants introduced after excavation and ancient contaminants acquired over many millennia of deposition can severely hinder efforts to conclusively identify authentic use-related residues. Accordingly, we propose, researchers must proceed from the null hypothesis that any residues observed on artifacts are *not* related to their use in the past. It must be presumed from the beginning that any adhered substances are contamination, either ancient or modern, and archaeologists must work to *disprove* that they are contamination. This, for example, is how to identified stearamide on the LKT1 artifacts. If, instead, one starts with an assumption that micro-residues are use-related, there is considerable risk for the misidentification of a contaminant as a use-related residue.

One particular focus here is that residues, like the assemblages themselves, are palimpsests, and the timespans involved in producing these palimpsests vary tremendously. The field of forensic science has formulated what is called Locard’s exchange principle, which is often summarized as “every contact leaves a trace” or “given contact between two items, there will be an exchange” [72]. According to this axiom, it should be expected that use-related residues should be gleaned via contacts between artifacts and foodstuffs, even if the artifacts’ actual contacts with fat or bone amounts to a total time that can be measured in seconds, minutes, or perhaps hours. Consider these contacts in light of others that such artifacts experienced. After their discard, the artifacts sat in sediments, not a sterile environment, for tens of millennia, the vast majority of their existence. After their recovery, one can imagine that a skillful and meticulous lithic illustrator might handle, scrutinize, and manipulate a particular artifact for longer than its owner employed it to actually slice through muscles and ligaments. The palimpsest of all these contacts cannot be ignored. It is crucial that, for researchers to claim that a specific residue is related to use, all other sources of contamination must be controlled for and ruled out one-by-one. A simple wash of an artifact will never be sufficient to address those issues that we document here.

## Supporting information

S1 File (ZIP)
