# Supplementary figures and images for: Every contact leaves a trace: Documenting contamination in lithic residue studies at the Middle Palaeolithic sites of Lusakert Cave 1 (Armenia) and Crvena Stijena (Montenegro)
Source: PLoS One. 2022 Apr 7;17(4):e0266362. doi: 10.1371/journal.pone.0266362 (PMC8989205; doi:10.1371/journal.pone.0266362)

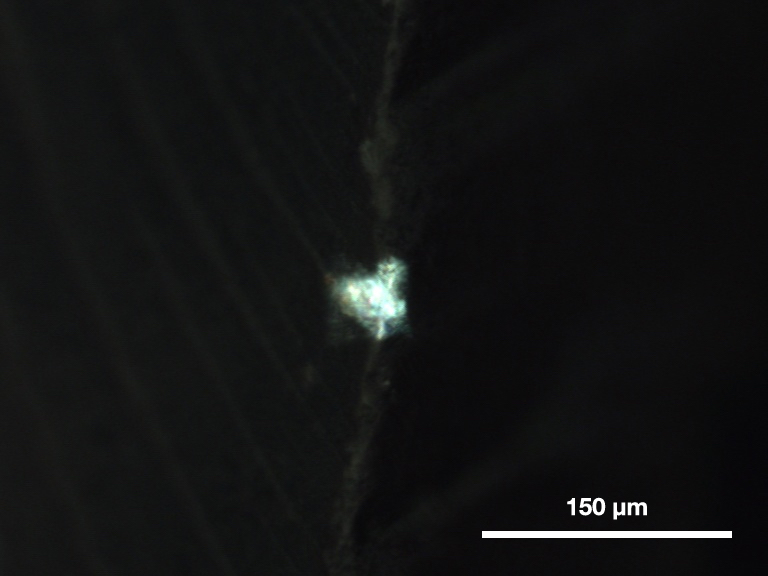

Supplement: S1 File — (ZIP) [file pone.0266362.s001.zip › S12 Fig for Fig 5 - image of LKT1 g04_1026_Spec_C.JPG]

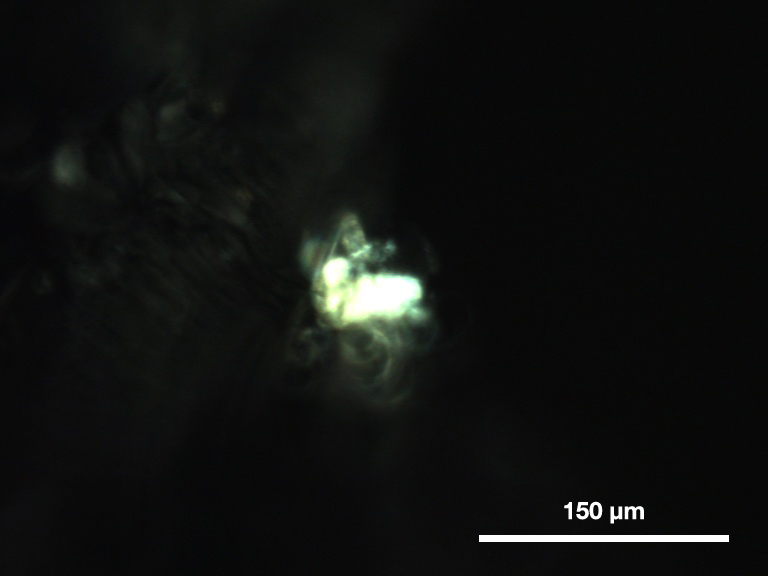

Supplement: S1 File — (ZIP) [file pone.0266362.s001.zip › S13 Fig for Fig 6 - image of LKT1 h05_699_Spec_D.JPG]

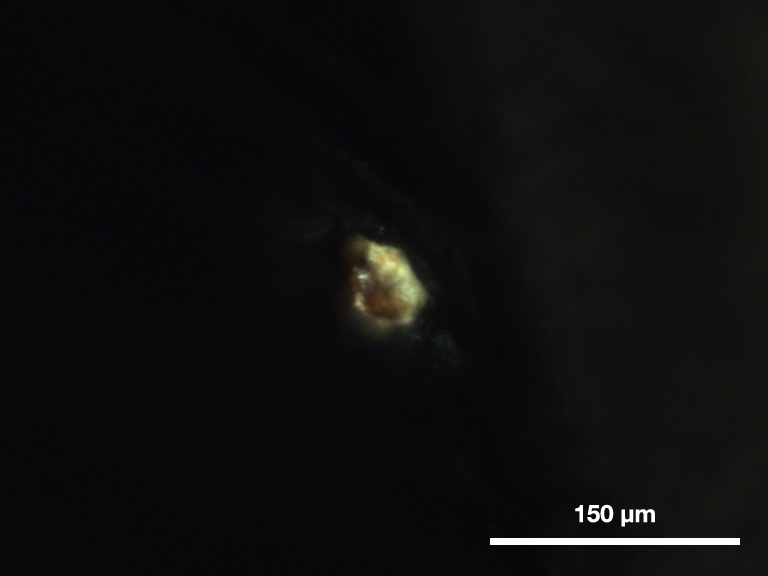

Supplement: S1 File — (ZIP) [file pone.0266362.s001.zip › S14 Fig for Fig 6 - image of LKT1 h05_699_Spec_E.JPG]

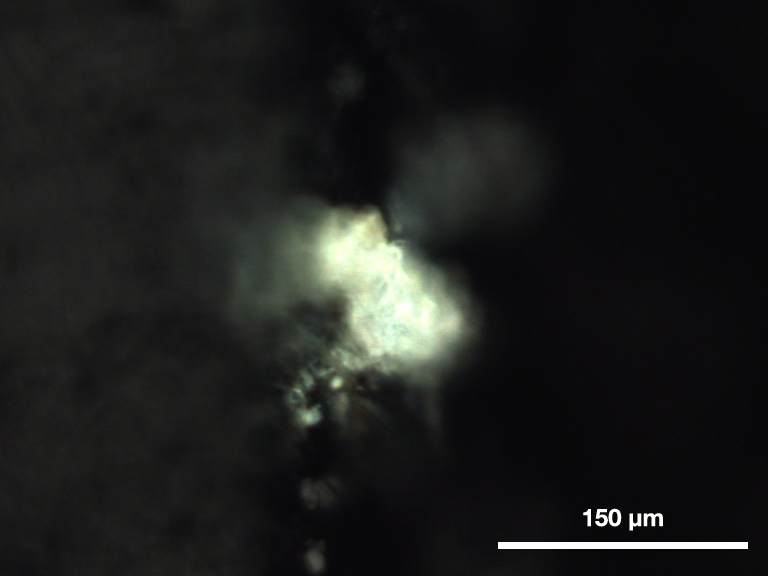

Supplement: S1 File — (ZIP) [file pone.0266362.s001.zip › S15 Fig for Fig 7 - image of LKT1 h05_442_Spec_B.JPG]

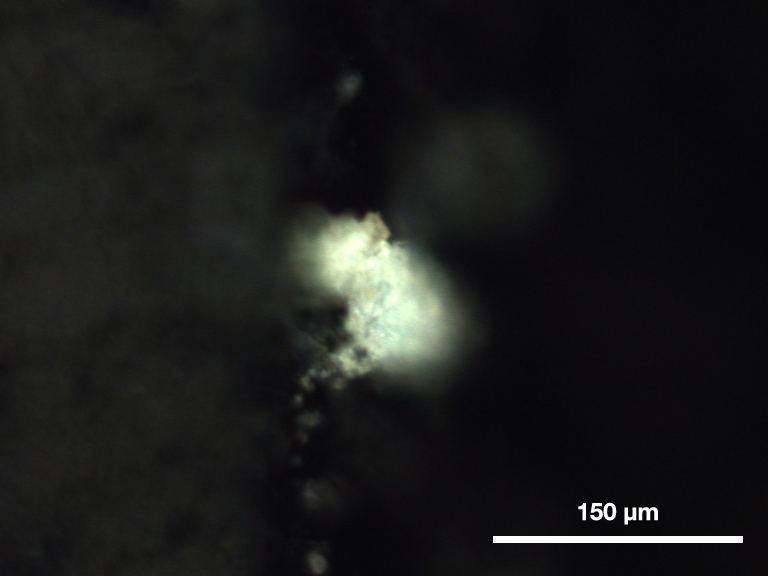

Supplement: S1 File — (ZIP) [file pone.0266362.s001.zip › S16 Fig for Fig 7 - image of LKT1 h05_442_Spec_D.JPG]

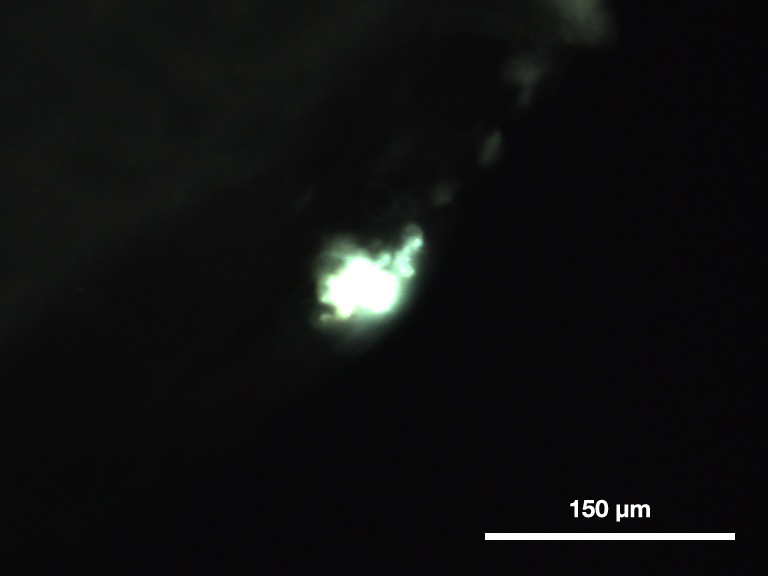

Supplement: S1 File — (ZIP) [file pone.0266362.s001.zip › S17 Fig for Fig 8 - image of LKT1 f05_891_Spec_H.JPG]

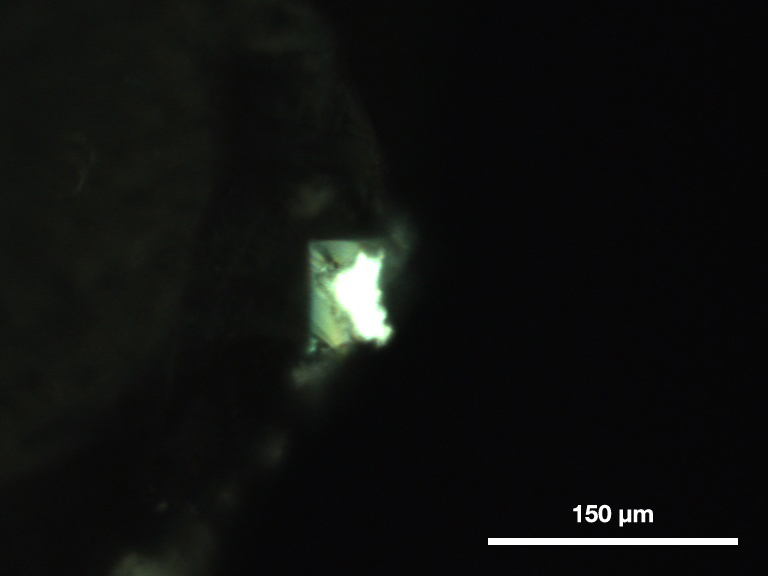

Supplement: S1 File — (ZIP) [file pone.0266362.s001.zip › S18 Fig for Fig 8 - image of LKT1 f05_891_Spec_J.JPG]

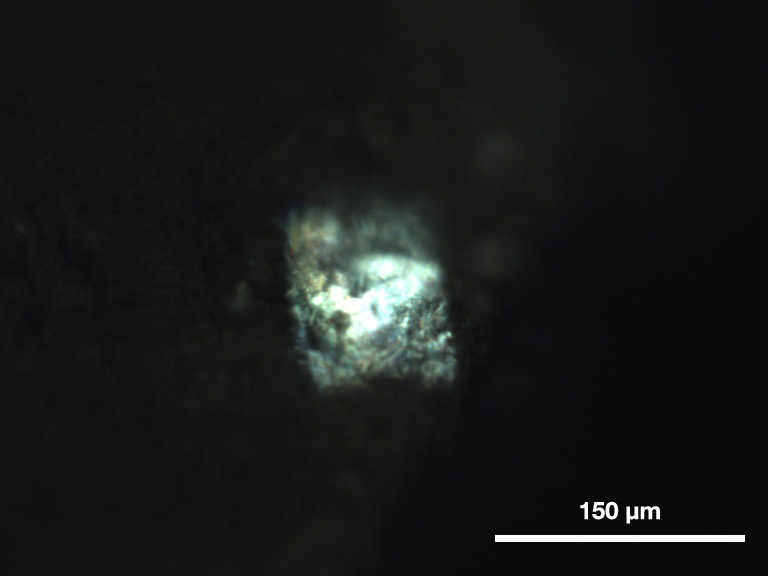

Supplement: S1 File — (ZIP) [file pone.0266362.s001.zip › S19 Fig for Fig 9 - image of LKT1 h05_477_Spec_I.JPG]

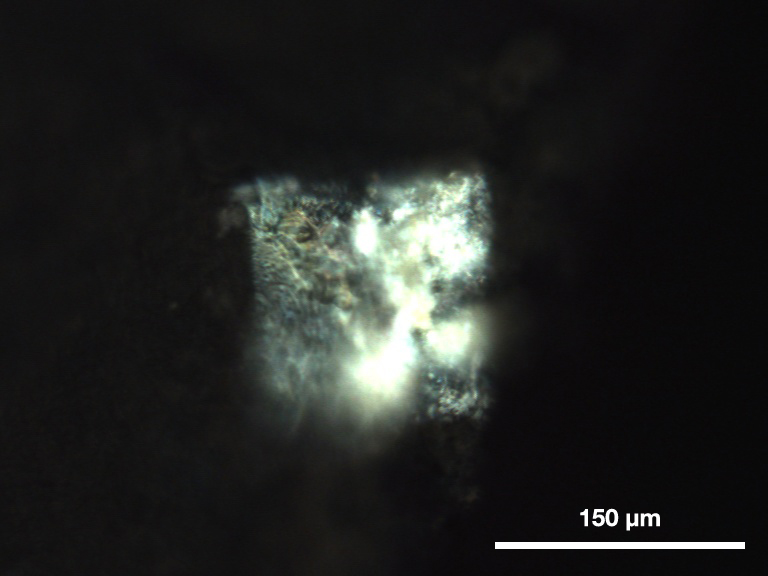

Supplement: S1 File — (ZIP) [file pone.0266362.s001.zip › S20 Fig for Fig 9 - image of LKT1 h05_477_Spec_K.JPG]
